# Machine Learning for Toxicity Prediction Using Chemical Structures: Pillars for Success in the Real World

**DOI:** 10.1021/acs.chemrestox.5c00033

**Published:** 2025-05-02

**Authors:** Srijit Seal, Manas Mahale, Miguel García-Ortegón, Chaitanya K. Joshi, Layla Hosseini-Gerami, Alex Beatson, Matthew Greenig, Mrinal Shekhar, Arijit Patra, Caroline Weis, Arash Mehrjou, Adrien Badré, Brianna Paisley, Rhiannon Lowe, Shantanu Singh, Falgun Shah, Bjarki Johannesson, Dominic Williams, David Rouquie, Djork-Arné Clevert, Patrick Schwab, Nicola Richmond, Christos A. Nicolaou, Raymond J. Gonzalez, Russell Naven, Carolin Schramm, Lewis R Vidler, Kamel Mansouri, W. Patrick Walters, Deidre Dalmas Wilk, Ola Spjuth, Anne E. Carpenter, Andreas Bender

**Affiliations:** † Broad Institute of MIT and Harvard, Cambridge, Massachusetts 02142, United States; ‡ Department of Chemistry, University of Cambridge, Cambridge CB2 1EW, U.K.; § Department of Pharmaceutical Chemistry, Bombay College of Pharmacy, Mumbai 400098, India; ∥ Department of Computer Science and Technology, University of Cambridge, Cambridge CB3 0FD, U.K.; ⊥ IgnotaLabs, Cambridge CB4 0GA, U.K.; # Axiom Bio, San Francisco, California 94107, United States; ⊗ UCB Pharma U.K., Slough SL1 3WE, U.K.; ● GSK, London WC1A 1DG, U.K.; ○ Novartis Biomedical Research, Cambridge, Massachusetts 02139, United States; ◆ Eli Lilly & Company, Indianapolis, Indiana 46285, United States; ◇ Relation Therapeutics, London NW1 3BG, U.K.; ■ Non Clinical Drug Safety, Merck Inc., West Point, Pennsylvania 19486, United States; □ AstraZeneca, Pepparedsleden 1 43183 Molndal, Sweden; ▲ Toxicology Data Science, Bayer SAS Crop Science Division, Valbonne Sophia-Antipolis 06560, France; △ Pfizer, Worldwide Research, Development and Medical, Machine Learning & Computational Sciences, Berlin 10922, Germany; ▼ Recursion, London N1C 4AG, U.K.; ▽ Computational Drug Design, Digital Science & Innovation, Novo Nordisk US R&D, Lexington, Massachusetts 02421, United States; ¶ Sanofi, Babraham Research Campus, Cambridge CB22 3AT, U.K.; α Eli Lilly and Company, Bracknell RG12 1PU, U.K.; β NIH/NIEHS/DTT/NICEATM, Research Triangle Park, North Carolina 27709, United States; γ Relay Therapeutics, Cambridge, Massachusetts 02141, United States; δ Nonclinical Safety, Collegeville Pennsylvania 19426, United States; ϵ Department of Pharmaceutical Biosciences and Science for Life Laboratory, Uppsala University, Uppsala 751 24, Sweden; ζ Phenaros Pharmaceuticals AB, Uppsala 75239, Sweden; θ College of Medicine and Health Sciences, Khalifa University of Science and Technology, Abu Dhabi 127788, United Arab Emirates

## Abstract

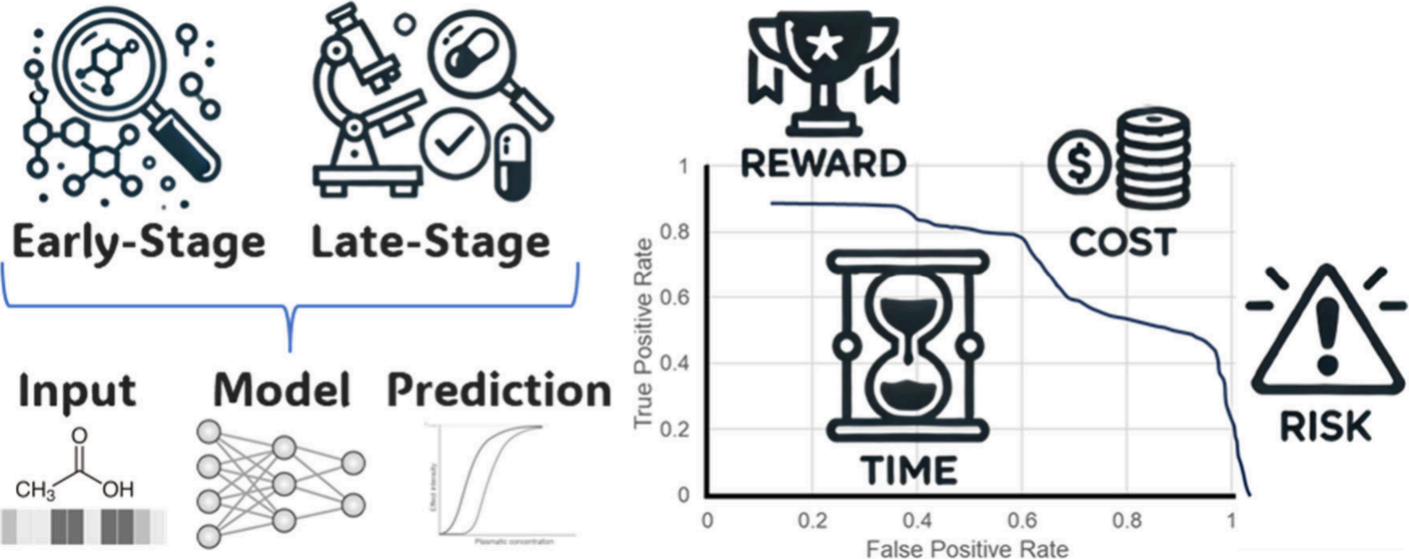

Machine learning (ML) is increasingly valuable for predicting molecular properties and toxicity in drug discovery. However, toxicity-related end points have always been challenging to evaluate experimentally with respect to *in vivo* translation due to the required resources for human and animal studies; this has impacted data availability in the field. ML can augment or even potentially replace traditional experimental processes depending on the project phase and specific goals of the prediction. For instance, models can be used to select promising compounds for on-target effects or to deselect those with undesirable characteristics (e.g., off-target or ineffective due to unfavorable pharmacokinetics). However, reliance on ML is not without risks, due to biases stemming from nonrepresentative training data, incompatible choice of algorithm to represent the underlying data, or poor model building and validation approaches. This might lead to inaccurate predictions, misinterpretation of the confidence in ML predictions, and ultimately suboptimal decision-making. Hence, understanding the predictive validity of ML models is of utmost importance to enable faster drug development timelines while improving the quality of decisions. This perspective emphasizes the need to enhance the understanding and application of machine learning models in drug discovery, focusing on well-defined data sets for toxicity prediction based on small molecule structures. We focus on five crucial pillars for success with ML-driven molecular property and toxicity prediction: (1) data set selection, (2) structural representations, (3) model algorithm, (4) model validation, and (5) translation of predictions to decision-making. Understanding these key pillars will foster collaboration and coordination between ML researchers and toxicologists, which will help to advance drug discovery and development.

## Introduction

In recent years, machine learning (ML) approaches for toxicity prediction using chemical structures and sometimes additional data sources have attracted widespread interest, particularly in drug discovery.^1^ There are constant innovations in ML for investigating biological systems and understanding their interactions with drugs, resulting in therapeutic activity and/or adverse outcomes. Still, these improvements must be reflected in practice. Currently, the costs of bringing a drug to market are increasing,^2^ while the overall success rates in clinical drug development remain poor.^3^ ML models can be trained on data from empirical assays to predict the properties of compounds from molecular structure (see Box 1 for standard definitions for ML related to predictive models for molecular toxicity). These models are often termed Quantitative Structure Activity/Property Relationship (QSAR/QSPR) models. By enabling the assessment and triage of compound toxicity prior to synthesis and physical testing, these approaches can streamline the drug discovery and development process. Rapid *in silico* screening procedures allow a larger number of potential candidates to be assessed in a shorter time and at decreased cost.

ML models can be used to predict compound properties including efficacy, toxicity (both on-target and off-target biology), and Absorption, Distribution, Metabolism, and Excretion (ADME) or Pharmacokinetic (PK) properties. On-target toxicity occurs when a drug affects its intended target and this itself causes adverse effects, while off-target toxicity arises from interactions of the drug with unintended biological targets.^4^ Certain PK properties and dosing schedules can help mitigate on-target toxicity by optimizing how the drug is absorbed, distributed, metabolized, and excreted, effectively controlling its exposure levels. Moreover, more potent compounds with favorable pharmacokinetic properties often requires smaller doses to achieve the desired efficacy. This reduction in the level of necessary exposure can decrease the likelihood of off-target toxicity, thereby improving the overall benefit-risk profile of the drug. Therefore, efficacy and PK/ADME properties drive target engagement and, thus, potential toxicity, all three need to be combined to arrive at a human-relevant toxicity estimation.

For ML-based QSAR/QSPR models, chemical structures of compounds are usually the basis for predictions of continuous (numerical) or categorical labels (properties) biologically relevant to toxicity.^5^ In addition to predictive models, recent advancements in generative models have emerged as powerful tools for designing novel chemical structures while optimizing for properties like low toxicity.^6^ This Perspective consolidates insights from both academia and industry on the application of ML models across various stages of drug discovery—ranging from early screening and alert systems in hit identification to compound optimization. We focus on discussing the role of ML models in predicting *in vitro* toxicity assays, *in vivo* animal study outcomes, and human-relevant toxicity throughout the drug discovery process.

## Challenges in Translating Preclinical Toxicity Data to Human-Relevant Predictions

In the early stages of drug discovery, High-Throughput Screens^7^ (HTSs) are commonly applied to screen extensive chemical libraries and find compounds with potential efficacy against a target or phenotype of interest (on-target effects), albeit with some arguments for^8^ and against^9^ its efficiency. Screens can be cellular or cell-free (isolated protein), with the former testing some aspects of permeability that the latter does not consider. After identifying hits, molecules are optimized to enhance their potency, selectivity, and ADME properties. Usually, *in vitro* assessment of toxicity, including off-target screens, are not conducted in a high-throughput manner; at early screening phases, unless there is an obvious off target activity, selective secondary-pharmacology screens are often run in multiple ‘waves’.^10,11^

In later stages of the drug discovery pipeline, *in vitro* human and *in vivo* animal data are used to predict human-relevant toxicity, which is the ultimate goal. However, though widely used, these have shown variable accuracy. For instance, *in vitro* assays have been reported to detect only 50–60% of rare and idiosyncratic drug-induced liver injury (DILI) cases in humans,^12^ while animal models often fail to fully capture human-specific toxicity due to species differences in drug metabolism and response.^13−16^ While studies in preclinical species can give insights into the drug’s effects on a whole organism, they have significant drawbacks. These *in vivo* models are also expensive and not scalable and raise ethical concerns. Therefore, there is a growing desire for alternative models that can more closely mimic human physiology. Complex models using human cells, such as *ex vivo* tissue slices and *in vitro* primary cells or spheroids/organoids, are increasingly used in an attempt to better predict human responses to drugs and decrease animal use in line with the FDA modernization act 2.0^17−19^ and related regulations worldwide. There is ongoing work on New-Approach Methodologies (NAMs) that avoid animals for early screening and gaining regulatory acceptance;^20^ over 250 regulatory-relevant NAMs have been proposed (https://nams.network/explore). These advancements align with the FDA Harmonization Act and the 3Rs (Replacement, Reduction, and Refinement) principles, which advocate for reducing the need for animal testing and instead opting for more ethical and scalable alternatives.^18^ Tissue slices, spheroid or organoid models are usually more physiologically relevant than traditional 2D cultured cancer-derived cell lines. For example, HepaRG, a human hepatic *in vitro* cell line, is widely used because it closely mimics primary human hepatocytes.^21^ These cells retain essential functions, such as major cytochrome P450 (CYP) enzymes, key phase 2 enzymes, nuclear receptors, and transporters, making them a valuable tool for studying hepatic drug metabolism and toxicity. However, they do not recapitulate the three-dimensional (3D) structure of the organ and some other aspects; hence a push toward the more expensive tissue slices or spheroids/organoids.^22^ Overall, progress in replacing animal testing with human-based NAMs has advanced significantly.^23^

Many toxicity hurdles that slow down the development process are detected in animal experiments rather than in the less-expensive initial screening stages.^24^ This underscores the urgent need for more reliable ML models for preclinical safety assessments. Since animal data remains a critical factor in determining a compound’s viability, predicting human findings using animal data will remain critical in the near term. Still, advancing toward methods that offer better understanding, more predictive power, and cost-effectiveness represents clear scientific progress. By integrating *in vitro* systems with advanced omics technologies and ML, there is potential to reduce the reliance on animal testing. The key challenge lies in recapitulating biological complexity to improve predictivity, which is needed for better decision making.^25^ Therefore, models that integrate PK/ADME with toxicity and predict *in vitro* to *in vivo* translation, or animal-to-human translation could accelerate drug discovery^26,27^ by incorporating interspecies and intersex toxicodynamic and toxicokinetic properties.^28,29^ Prioritizing such translational models may bridge the gap between *in vitro* or animal data and human data, enabling faster, cost-effective development of safer and more effective medicines.

## Guidelines for ML-Based Toxicity Models

For the use of ML-based models in drug discovery, model reliability is key. Deprioritization of compounds with undesired properties and progression of those without any early risk flags requires decision-making based on ML models. To ensure the credibility of QSAR/QSPR models, the Organisation for Economic Co-operation and Development (OECD) has defined five principles for model validation.^30,31^ These include: (i) a defined end point, (ii) an unambiguous algorithm, (iii) a defined domain of applicability (in terms of chemical structures), (iv) appropriate measures of goodness-of-fit, robustness, and predictivity, and (v) a mechanistic interpretation, if possible. These five principles serve as a foundational framework for evaluating the quality and reliability of QSAR/QSPR models in regulatory contexts, ensuring that predictions are applicable to real-world scenarios. A complementary set of four guidelines was recently proposed for using ML models for ADME/PK for small molecule lead optimization.^32^ They include frequently retraining models using data sets from multiple sources (global data) as well as new experimental data (local data), and ensuring models are interactive, interpretable, and integrated into chemists’ tools. Bender et al. propose a general set of guidelines for the development and evaluation of ML tools, particularly focusing on supervised learning.^33^ Their guidelines emphasize the importance of comprehensive data reporting, conducting retrospective evaluations, comparing models against baselines, performing prospective testing, and ensuring a thorough model interpretation. Additionally, they provide specific recommendations for reporting standards and evaluation metrics, aiming to enhance the reliability and applicability of ML models in addressing real-world chemical challenges.

The risks of reliance on ML include the potential for inaccurate or mis-calibrated models to select drug candidates with undesirable properties, including safety risks. Mis-calibration, here, refers to when the model predicted probabilities do not accurately reflect the true likelihood of observed outcomes. Mis-calibrated models can be a result of (a) low information content (Box 1) in the feature space being used to train models, (b) exposure to narrow chemical space in training data, which then leads to a narrow applicability domain (because of overfitting of models to a small chemical space), or (c) improper (and often nonexistent) prospective validation of models (for future projects), which can lead to overoptimizing and deployment of models that do not generalize.

**Chart Box 1:**
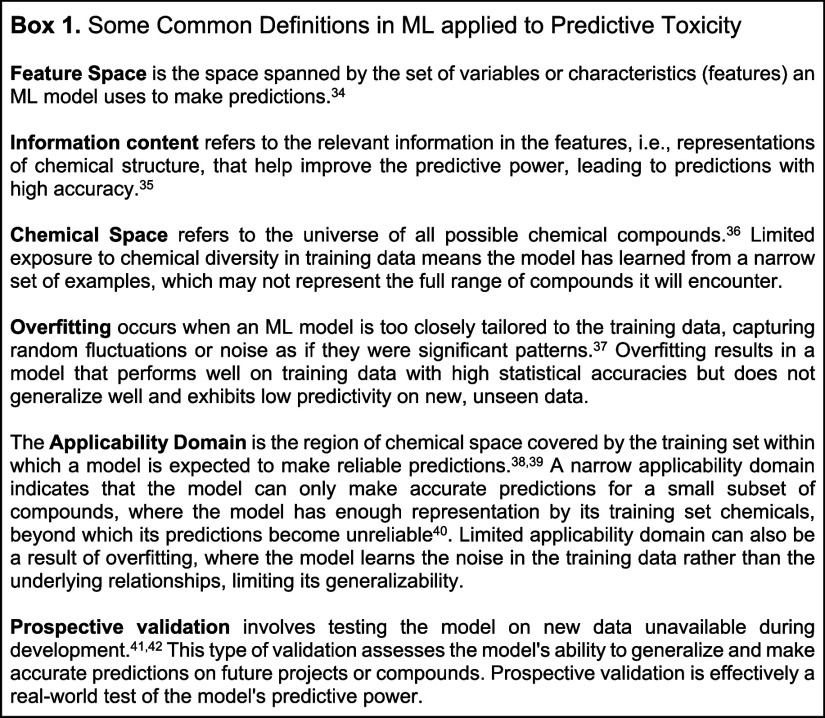


**Figure 1:**
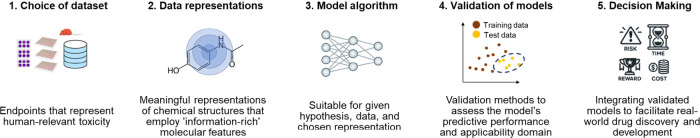
Five critical pillars deserving attention from researchers using ML tools for toxicity prediction are discussed in this review. Pillar 1: Choice of data end points, data sets, and data preparation.

Building on these principles in the context of ML-driven drug discovery, in this work, we propose five critical pillars for success using ML tools for toxicity prediction (Figure 1):

1. Selecting appropriate data sets that accurately represent the toxicity of interest ensures that model predictions are relevant.
2. Chemical structures must be encoded into relevant representations that capture essential molecular ‘information’ to generate ML-ready features.
3. Model algorithms must be suitable to learn the signal in the data and representation characteristics mentioned above.
4. Models must be validated to assess their predictive performance, both retrospectively and prospectively, within their applicability domain.
5. In practical model applications, it is crucial to consider project scenarios and desired outcomes to facilitate real-world drug discovery and development.

Adopting these five pillars would enhance the translatability and reliability of predictive models in toxicity prediction, potentially accelerating the design of safe and effective therapeutic candidates. In the subsequent sections, we provide a detailed examination of these five pillars.

**Figure 2:**
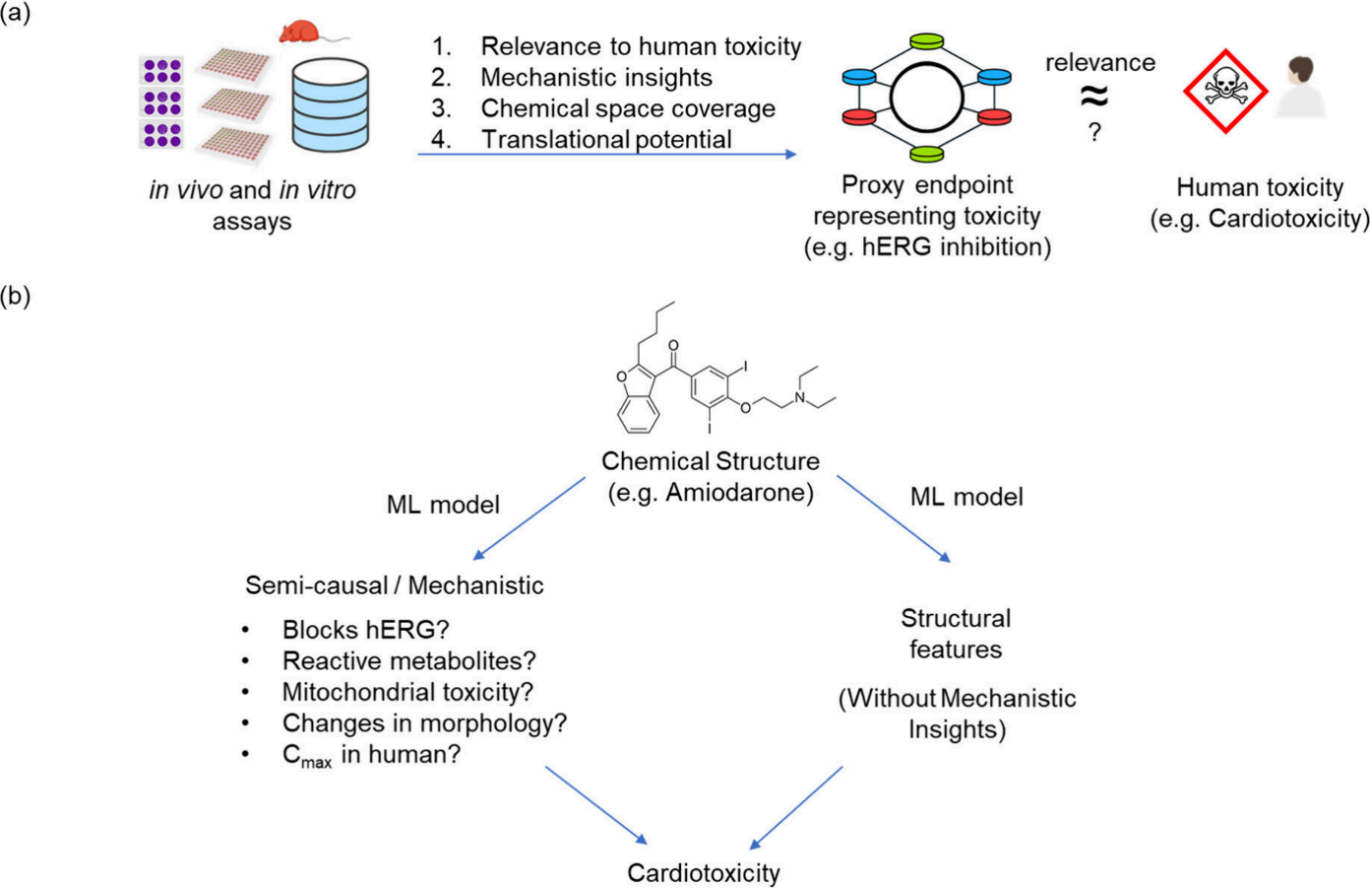
(a) The general criteria when choosing the *in vitro* or *in vivo* assay as a proxy end point that could represent human toxicity learned by an ML model. (b) The choice of proxy end point will influence what the model learns and whether mechanistic insights are possible.

When predicting toxicity, there is typically limited data available for the specific end point of interest. Although human toxicity prediction is the ultimate goal, animal studies are more common than in humans and can provide granular information, including dose response, time response, and target organ characterization. Recently, one of the largest data set with *in vivo* toxicity data for 80,000 compounds against a total of 59 acute systemic toxicity end points was analyzed by Sosnin et al. and later made publicly available by Jain et al.^43,44^ However, *in vivo* data is only available at advanced stages of compound characterization and *in vitro* data is used for early screening. Thus, *in vitro* assays correlate with one or multiple aspects of the end point of interest, but *they are not the* in vivo *relevant end point itself*. Due to data availability, training data sets for ML models very often include data from such experimental *in vitro* assays that typically proxy for a human clinical end point (Figure 2a). Where mechanisms are better understood, well-studied proxy assays can be used, hence leading to both better predictivity and better interpretability (Figure 2b).^45,46^ For example, these could involve functional or binding assays for proteins involved in known mechanisms of toxicity (for example, human ether-a-go-go-related, hERG channel inhibitors in cardiotoxicity^47^ or imaging screens for organelle toxicity such as mitochondrial membrane depolarization^48,49^). Hence, the situation differs from case to case quite significantly, where better mechanistic understanding is present different (e.g., target-based) assays might be employed; but in either case the prediction of *in vivo relevant toxicity* is the goal (as opposed to just assay labels), in order to arrive at a prediction in the end that can be used for real-world decision making.

## Choosing Relevant (Predictive) Assay End Points for *In Vivo* Toxicity

An ideal proxy end point strongly predicts the phenotype observed in humans. The design or selection of proxy end points requires careful consideration to ensure reliability, translatability, and relevance to the application area the model’s predictions aim to serve.^50^ For example, an assay commonly used to gauge a common cardiotoxicity mechanism detects Human Ether-a-Go-Go-Related Gene (hERG) channel inhibitors. However, the hERG assay is merely a proxy end point for some cardiotoxicity signals such as prolonging the QT interval of ECG in a beating heart.^51^ It does not convey the potential severity of the induced cardiotoxicity by itself; rather, the extent of QT prolongation (extended interval between the heart contracting and relaxing) and the risk of progressing to TdP (Torsades de Pointes, a type of atypical heart rhythm) vary significantly among compounds known to inhibit hERG (even at very similar activity against hERG itself), illustrating the importance of taking into account the ADME properties of drugs in toxicity prediction.^52^ Some proxy end points have been very well established, such as skin sensitization for cosmetics, where the European Union (EU) has already phased out animal-based experiments.^53^ Key Events (KEs) are well-defined for skin sensitization, which often involves chemical reactivity, offering detailed mechanistic insight into how chemicals trigger skin sensitization. The OECD has specified a series of *in vitro* assays to accurately measure many of the early key events in skin sensitization. For instance, KE1, which involves the covalent binding of haptens to skin proteins, is effectively assessed by the Direct Peptide Reactivity Assay (DPRA), Amino Acid Derivative Reactivity Assay (ADRA), and the kinetic DPRA assay, and these are well-established methods from the OECD.^54^ Proxy end points such as activity against protein targets are mechanistically understood and help gather information on the bioactivity profile of the compounds (Table 1). Secondary pharmacology (off-target effect) is usually screened for using a panel of targets with a relatively well-established relationship to human toxicity (for details, see Bowes et al.^11^ and Brennan et al.^55^).

**Table 1:** Commonly Used *In Vitro* Assays That Can Be Used as Proxies for Human-Relevant Toxicity End Points^a^

Toxicity	Assays for Proxy End points	Comment	Commonly Used Data sets in Published Predictive Models
Hepatotoxicity	HepG2 cell viability assays, CYP inhibition assays,^62^ mitochondrial membrane depolarization, transporter and reactive metabolite assays	Assess liver function and potential for liver damage through cell viability and enzyme inhibition	Gates Library screen for HepG2 cell viability,^63^ CYP P450 Inhibition^64^
Cardiotoxicity	Human induced pluripotent stem cell-derived cardiomyocytes (hiPSC-CMs) assays,^65^ hERG, Nav1.5, and Cav1.2 assays	Evaluate effects on cardiac cells and potential for arrhythmia through cardiac cell function and ion channel activity	qHTS for Inhibitors of hERG,^66^ hERGCentral^67^
Nephrotoxicity	Kidney proximal tubule cell assays,^68,69^ HK-2 cell cytotoxicity assays^68,69^	Assess kidney cell damage and function, focusing on tubule cells and cytotoxicity	*in vitro* models based on HK-2 cells^70^
Neurotoxicity	Neuronal cell viability assays,^71,72^ Neurite outgrowth assays,^71,72^ and RNAi screen for *Drosophila* neurons	Evaluate potential damage to neurons and effects on neurite growth, indicating neurotoxicity	*in silico*, *in vitro*, and *in vivo* end points of neurotox including sedation, ataxia, and seizure detection^73^
Genotoxicity and Carcinogenicity	Ames test, Micronucleus assay,^74^ Comet assay, Cell transformation assays^75−79^	Assess DNA damage and mutagenic potential, indicating genetic toxic risks. Evaluate the potential for causing cancer through cell transformation and chromosomal damage	Ames data set collated by Xu et al. (80) CCRIS (Chemical Carcinogenesis Research Information System) Data^81^
Endocrine Disruption	Reporter gene assays (e.g., for estrogen receptor activity), H295R steroidogenesis assay^82,83^	Assess interference with hormone activity, focusing on receptor binding and hormone production	Endocrine-related *in vitro* assays from ToxCast^84^
Skin Sensitization	Local lymph node assay (LLNA),^85^ guinea pig maximization test (GPMT)	Evaluate the potential for causing skin irritation or damage using human skin models	AOP-related assays in SkinSensDB^86^– KeratinoSens/LuSens, human Cell Line Activation Test (h-CLAT), Local lymph node assay (LLNA)
Ocular Toxicity	Bovine corneal opacity and permeability (BCOP) assay, HET-CAM assay^87^	Assess the potential for causing eye irritation or damage through corneal and membrane assays	GHS classifications based on Draize rabbit eye test^88^

a Data from these assays are commonly used in the ML models. For more details on the secondary pharmacology panel of targets, see Bowes et al.^11^ and Brennan et al.^55^

Selecting an end point to be predicted goes hand in hand with selecting data sets containing values for that end point. Companies have their own in-house toxicity data and there are also publicly available data sets, such as reviewed in Vo et al.,^56^ Cavasotto et al.,^57^ Huang et al.,^58^ and Schapin et al.^59^ Regardless of the source, data sets often suffer from technical issues that require careful preprocessing and data curation. For instance, there might be invalid chemical structures, inconsistent chemical representations, or undefined stereochemistry. Moreover, data sets might include irrelevant toxicity end points or high assay artifact rates, which do not allow for proper ML benchmarking.^60^ Here, we hence present a list of data sets structured by the type of organ-based toxicity they aim to predict (Table 2) that were carefully examined and represent clearly defined end points, with outcomes that are likely to be reproducible. Data sets such as the Novartis Secondary Pharmacology Database (SPD)^61^ and Biogen ADME^41^ provide a large number of data points from consistently measured state-of-the-art *in vitro* assays and commercially available compounds, which might be particularly useful for newcomers seeking to understand the available data landscape when building and benchmarking ML models. Nevertheless, the precise use of a data set for a particular purpose requires case-by-case consideration and can hence not be generically answered here.

**Table 2:** Data Sets Examined by Authors of This Study That Are Recommended for Machine Learning Models as They Represent Large Data Sets with Well-Defined End Points

Data Set	Type of Data (*in vivo*/*in vitro*)	Data Type in Original Publication	Data Points and End Point	Comments	Link to Data Set	Source
Novartis SPD	*in vitro* - targets	Quantitative/Numerical	1958 drugs assayed for 200 secondary pharmacology targets linked to ADEs; 150k AC_50_ values with 121k unique entries; Cmax(free) for 940 drugs; Dose–response measurements	Internal data (*in vitro* safety pharmacology assays)	https://zenodo.org/records/7378746	(61)
Biogen ADME	*in vitro* - ADME	Quantitative/Numerical	3521 pharmaceuticals; 6 *in vitro* ADME end points–Human and rat liver microsomal stability, MDR1-MDCK efflux ratio, solubility, and human and rat plasma protein binding assays	Internal prospective data sets (six ADME *in vitro* end points over 20 time points)	https://github.com/molecularinformatics/Computational-ADME	(41)
Activity Cliff Eindhoven	*in vitro* - Activity Cliff/Bioactivity	Qualitative/Categorical	48,707 molecules for 30 targets, including kinases, nuclear receptors, G-protein-coupled receptors, transferases, and proteases	Curated data on 30 macromolecular targets from ChEMBL; Nonproprietary; Small molecules	https://github.com/molML/MoleculeACE	(89)
Activity Cliffs Zhang	*in vitro* - Activity Cliff/Bioactivity	Qualitative/Categorical	400 K Matched Molecular Pairs (MMPs) against 190 targets (142,307 activities), including over 20K MMP-cliffs and 380 K non-AC MMPs	Collected from publicly available database ChEMBL; Nonproprietary; Available to Buy; Small molecules	https://drugai.github.io/ACNet/	(90)
Activity Cliffs Morris	*in vitro* - Activity Cliff/Bioactivity	Quantitative/Numerical	Three binding affinity data sets testing for small molecule inhibitors: 8883 compounds for dopamine receptor D2; 3605 compounds for factor Xa; 1924 compounds for SARS-CoV-2 main protease	Collected from sources: ChEMBL compounds with *K*_i_ values for DRD2 and FXA, Covid Moonshot IC50 compounds from COVID moonshot	https://github.com/MarkusFerdinandDablander/QSAR-activity-cliff-experiments	(91)
AZ ADMET - Clearance (Hepatocytes)	*in vitro - Pharmacokinetic*	Quantitative/Numerical	408 compounds with intrinsic clearance measured in human hepatocytes; 837 compounds with intrinsic clearance measured in rat hepatocytes	Internal Experimental data determined at AstraZeneca	https://www.ebi.ac.uk/chembl/assay_report_card/CHEMBL3301372/, https://www.ebi.ac.uk/chembl/assay_report_card/CHEMBL3301371/	(92)
AZ ADMET - Clearance (Microsomal)	*in vitro - Pharmacokinetic*	Quantitative/Numerical	1102 compounds with intrinsic clearance measured in human liver microsomes	Internal Experimental data determined at AstraZeneca	https://www.ebi.ac.uk/chembl/assay_report_card/CHEMBL3301370/	
AZ ADMET - PPB	*in vitro - Pharmacokinetic*	Quantitative/Numerical	1614 compounds with percentage bound to plasma by equilibrium dialysis	Internal Experimental data determined at AstraZeneca	https://www.ebi.ac.uk/chembl/assay_report_card/CHEMBL3301365/	
Genetox (Ames/IVMN) - US EPA	*in vitro* - Genetox	Qualitative/Categorical	8442 compounds; measures presence of *in vitro* micronucleus (IVMN)	Compiled from several public sources (e.g., TOXNET, COSMOS, eChemPortal)	https://github.com/g-patlewicz/genetox	(93)
Genetox (Ames/IVMN) - US EPA	*in vitro* - Genetox	Qualitative/Categorical	211 Ames negative, 682 Ames positive and other equivocal and *in vitro* micronucleus (IVMN) measurements	Compiled from several public sources (e.g., TOXNET, Carcinogenicity and Genotoxicity eXperience (CGX) database)	EURL-ECVAMNegative Ames - https://www.sciencedirect.com/science/article/pii/S1383571820300693?via%3DihubPositive Ames - https://data.europa.eu/data/datasets/jrc-eurl-ecvam-genotoxicity-carcinogenicity-ames?locale=en	(94,95)
Ames - ISSTOX	*in vitro* - Genetox	Qualitative/Categorical	5536 compounds evaluated for Ames test	Compiled from public data sets	https://data.mendeley.com/datasets/ktc6gbfsbh/2	(96)
Ames - Benchmark Data Set for *in silico* Prediction of Ames Mutagenicity	*in vitro* - Mutagenicity	Qualitative/Categorical	6512 compounds evaluated for Ames test	Compiled from several public sources (e.g., CCRIS, VITIK, GeneTox)	https://pubs.acs.org/doi/10.1021/ci900161g	(97)
Ames -*in silico* Prediction of Chemical Ames Mutagenicity	*in vitro* - Mutagenicity	Qualitative/Categorical	8348 compounds evaluated for Ames test	Compiled from several public literature sources	https://pubs.acs.org/doi/10.1021/ci300400a	(80)
Prenatal developmental toxicity	*in vivo* - prenatal developmental toxicity	Qualitative/Categorical	Chemicals with developmental toxicity classifications.	Compiled from public repositories and literature sources	https://pubs.acs.org/doi/10.1021/acs.est.2c01040?goto=supporting-info	(98)

## Chemical Diversity and Applicability Domain

The diversity of the represented chemical space is a key aspect to consider when choosing a data set for modeling. The extrapolation of ML models to novel chemistry (not observed during model training) is difficult to achieve in many cases, due to the size of chemical space available and the “local” behavior of properties for highly similar compound. Therefore, the data set for the selected proxy end point should ideally cover a wide range of chemical structures on which the model might be used. For this reason, models are often regularly updated by using assay results from the latest round of project compounds.

A local model built with a small data set lacking structural diversity will have a narrow applicability domain, i.e., strong performance only within a particular area of chemical space. Nevertheless, this might be very useful for a particular project. Local models typically have poor performance on novel chemical scaffolds; that is, scaffolds that represent different features from the chemicals used for training a model.^99,100^ Generally, most ML models predicting toxicity (but also bioactivity and other domains) are in practice local models with limited applicability in the larger chemical space, and extrapolation can only be expected to a limited extent due to the high dimensionality of input space and limited data.^101,102^ In theory, with large and diverse data sets, ML models ought to have stronger performance and generalizability. Such models are referred to as global models, and are typically trained with data from different series or scaffolds.^100^ However, despite their conceptually greater generalization potential, global models for toxicity prediction are often infeasible. One reason is data availability or large experimental variability as data sets are often published with inadequate metadata to be able to effectively combine measurements from different laboratories and/or with different equipment. One example is hERG inhibition, where we often see a massive variability in measured IC50 values due to how compounds were measured.^103^ Despite this variability, many charged hERG blockers share a basic nitrogen pharmacophore, which classification models—commonly used for this end point—can recognize when predicting across a broad chemical space.^104,105^ Global models work only in cases where the data available is sufficiently large and where the relationship of the end point with chemical structure is sufficiently simple for a global model to capture predictive trends (in the given descriptor space employed, see next section). For example global models may work better in the prediction of logD where large data sets are available^106,107^ (compared to models predicting toxicity outcomes). LogD is in principle largely additive in nature; that is, the same group on different scaffolds makes similar contributions (which then enables the model to extrapolate to new cases). Mannhold et al. observed RMSE = 1 for prediction of logP/logD7.4 for over 95,000 Pfizer compounds and mentioned this result as failure.^108^ The time split validation model for logD in the article reported an RMSE of 0.92 and is still worse than the 0.3 expected experimental error of this property. End points that are correlated with logD also show some additivity, but for examples where lipophilicity is not the driver of behavior (e.g., high logD, low unbound intrinsic clearance), usually ML models tends to predict incorrectly. A drug’s biological activity (potency), in contrast, tends to be more specific to the overall chemical structure (due to the dependency of bioactivity on a particular spatial arrangement of features in a protein binding pocket), and while it has a lipophilicity component, it is more often influenced by nonadditive effects from group combinations and shows less additivity across targets. Validating models within their applicability domain (and establishing this applicability domain in the first place) is an important topic that is discussed later in this work.

## Standardizing Chemical Structures

Preprocessing chemical data should include a clearly outlined and reproducible method to standardize molecules and generate ML-ready representations,^109^ considering validity, stereochemistry, solvents and counterions, tautomerism, and protonation states. Each of these factors plays a critical role in the molecule’s interaction with biological targets and also for the descriptors that are generated from the chemical structures as input for ML models.

Enantiomers can have vastly different pharmacokinetic and pharmacodynamic properties, impacting efficacy and safety. For example, (R)- and (S)-fluoxetine show differences in metabolism, receptor binding, and side effects.^110,111^ At the biological receptor level, enantiomers can also show stereoselectivity; d-asparagine tastes sweet whereas l-asparagine does not.^112^ At an experimental level, the presence of multiple enantiomers within a single screening compound sample reduces the potential concentration of active species and complicates the identification of hits from the assays. Separating the activity of various isomers from a racemic mixture is difficult because individual enantiomers must be tested to find which causes the true biological activity. At the modeling level, some molecular fingerprint representations do not encode stereochemistry, which needs to be taken into account (see next section). Moreover, in public databases, stereochemistry annotation is usually poor and absolute configuration is often unknown,^113^ particularly for natural products. Unfortunately, in our experience, where stereochemistry information is present, some of it is often arbitrarily chosen, as enantiomers may get separated early in preprocessing. One common approach in modeling is to remove stereochemistry information altogether before generating representations, and as most drug molecules are usually not chiral, this approach generally retains information content of the majority of the training data set (losing information only where stereochemistry has an impact on the respective end point). Unfortunately, some processing workflows involve selecting a single, arbitrary enantiomer, which is clearly not optimal. Another approach in modeling is determining all stereocenters, correcting invalid stereochemistry, and generating canonical isomeric SMILES that retain stereochemistry.^114,115^ In cases where stereochemistry has an impact on the respective end point, and where sufficient data is available for the model to learn this relationship, this approach may sometimes be advantageous.

Tautomers are structural isomers that are readily interconvertible due to the transfer of one or more protons.^116^ Tautomerism can explain certain bioactivity, for example, the presence of a double bond at the α,β-position adjacent to the keto carbonyl in α-ketoamide derivatives allows the formation of inactive tautomers that exist in equilibrium, and this leads to reduced efficacy against Dengue virus proteases.^117^ Thus, considering tautomers is important from a modeling perspective, as they vary in physicochemical properties such as logP, hydrophobicity, and solubility. Many attempts to predict low-energy tautomers have been unsatisfactory.^118^ The standardized molecule from tautomer enumeration will typically not represent the “correct” form in the solution, and exhaustive searches can yield nonphysical forms that cannot exist in the solution. The best approach in a standardization pipeline, in practice, can often be to be consistent throughout because often being *consistent* is more important than being *correct* for subsequent model generation steps. Significant efforts have been made in cheminformatics to study tautomerism in large data sets, in particular, on selecting the most consistent tautomer and capturing their molecular descriptors.^119−121^ Tools like RDKit and others provide methods to enumerate tautomers; however, they often handle tautomerism differently, and not all types of tautomerism are implemented in every software package, leading generally to different results. For example, PubChem standardizer generates a canonical tautomer during compound standardization, but ChEMBL does not.^114,115^ Overall, tautomers are often not trivial to handle when preprocessing structures for predictive modeling.

The protonation state of a molecule affects, among other biological properties, binding to transporters and enzymes, permeability across biological membranes, and pharmacokinetic and pharmacodynamic properties.^122^ Due to effects on distribution of a molecule, protonation state may be even more important for toxicity prediction than other end points. For example, drugs such as proton pump inhibitors, with a p*K*_a_ between 3.8 and 4.9, selectively accumulate in the acidic secretory canaliculi of parietal cells and are converted into active forms only under specific protonation conditions.^123^ When predicting pharmacokinetic parameters, a relevant pH must be considered for protonating compounds during compound standardization (such as the pH of blood, or the pH in the GI tract in case of absorption, etc.).^124,125^ The impact of protonation on featurization varies according to the descriptor used. For example, MACCS keys (Molecular ACCess System) have a key (key no. 49) that indicates the presence or absence of charge, while Morgan fingerprints are invariant to protonation states. Descriptors such as the Hammett electronic parameter (σ), logD, and the Taft constant are also impacted by the protonation state of the molecule. Hence, protonation states of molecules are important, but challenging aspects of preparing small molecules for modeling.^126^

## Checking End Point Data for Inconsistencies or Ambiguities

The next stage of preparing data for modeling involves assessing the biological data (end point space). Often more so than traditional ML data sets (for example, from the image or speech domain), biological data sets are conditional on experimental factors such as compound concentration, cell type, time points, and *in vivo* pharmacokinetics.^127^ Therefore, data from such end points require careful curation to remove inconsistencies or ambiguities in the measured end points and minimize the risk of introducing biases into ML models. For example, the Kendall Tau test can measure the ordinal association between two measured quantities, helping to identify inconsistencies in ranked data.^128^ For example, Landrum et al. demonstrated that in nearly 65% of instances, multiple IC50 assays for the same compound-target pair showed discrepancies of over 3-fold, with 27% of cases differing by more than 10-fold, and a Kendall’s Tau correlation of 0.51 between multiple assays measuring the same compound against the same target.^129^ Outlier detection methods, such as *Z*-scores or the Tukey IQR method, can highlight anomalies that may indicate errors.^130^ For example, Kalliokoski et. al recommends if discrepancies are observed in ΔpIC50 are greater than 2.5, there is a high probability they may contain errors and those inconsistent experimental records can be removed before modeling.^131^ For example, models designed to predict specific toxicities, such as mitochondrial membrane depolarization or DNA damage from *in vitro* assays, often inadvertently capture and predict broader indicators of overall cell viability instead.^132^ Such models may have been trained without confirmatory assay data—which combines results from a screening assay with a cytotoxicity counter screen to exclude highly cytotoxic compounds.^133,134^ Some modeling methods, such as Huber regression,^135,136^ are less sensitive to outliers, and given the noise in biological data, these can be preferable (an analogy being that median-averaging is less sensitive to outliers than mean-averaging). Other models, such as Probabilistic Neural Networks,^137^ can take large variations into account while training, and can generate predictions along with confidence intervals or prediction ranges that reflect the uncertainty or variability in the data. Overall, curating the biological end points to avoid biases or inaccuracies that could compromise model performance is essential for ensuring that models produce meaningful and actionable predictions.

## The Conditional Nature of Data and Categorization

A given compound is not simply “toxic” or “non-toxic”, as famously quoted by Paracelsus in 1538, “What is there that is not poison? All things are poison, and nothing is without poison. Solely the dose determines that a thing is not a poison”.^138^ The effective dose, Cmax (maximum concentration in the blood), and concentrations in relevant organs can vary by orders of magnitude depending on a molecule’s efficacy and its ADME properties.^139^ Therefore, any measurement of toxicity—whether through a simplified binary classification or a detailed dose–response curve—is useful only when it reflects clinically relevant doses. Given that toxicity (as other effects) can vary significantly depending on the experimental conditions, such as the dosage, duration of exposure, and the biological model used (organism, sex, target tissue, etc.), this ‘conditionality’ of biological data often represents a problem when training predictive models; it is rarely practical to capture experimental conditions in the model itself, given the sparsity of data across experiment variables.

Converting toxicity data sets into simplistic binary prediction problems can hence simplify ML strategies but serve as poor representations of biological realities. For example, selective estrogen receptor modulators stimulate estrogen receptors at very low (picomolar) concentrations, but at much higher (micromolar) concentrations, they impact microtubules, leading to a distinct activity profile.^140^ Binarization of continuous values leads to loss of information, especially for compounds that lie close to the threshold for binarization.^127^ Mervin et al. presented a compelling case for employing particular modeling methods to better use binary labels based on readouts that were originally continuous in nature, given reasonable constraints upon modeling objective and data sets.^141^ The presence of activity cliffs (where small changes in chemical structure lead to major changes in bioactivity) in data sets makes the distribution of labels highly heteroscedastic (uneven over the range of values measured). Given the unequal distribution of measurement error across the range of activity values, the authors argue that regression is also not favorable for *in silico* target prediction and suggest using Probabilistic Random Forest models. These models were shown to improve bioactivity predictions close to the classification threshold by taking into account the experimental uncertainty. To avoid choosing a single particular threshold for binary classes, one could incorporate multiclass categories such as “highly toxic”, “toxic”, “nontoxic”, and “ambiguous”. Multiclass categorization can use dose, exposure, or margins to set thresholds for classification calls. For example, models using *in vivo* toxicity data might set a threshold of total *C*_max_ value less than 10 μM but greater than 1 μM, which helps to set the context around the category. Representing intermediate activity or ambiguity can be particularly valuable in capturing the complexities of biological data and preventing the misclassification of borderline cases.

To summarize Pillar 1, the chosen data set must be relevant to the toxicity one wants to predict, represent chemical space sufficiently diverse and relevant for the intended use case, be high in information content, provide mechanistic insights (ideally), and be appropriately preprocessed to remove inconsistencies. Well-curated data (with a standardized framework for organizing and categorizing information where possible) reliably train models only when the encoded features capture the most relevant chemical and biological properties. Together, these elements enable the development of accurate, reliable, and generalizable predictive models that are more likely to be applicable in a real-world drug discovery context.

## Pillar 2: Encoding Chemical Structures into Information-Rich and/or Meaningful Features

**Figure 3:**
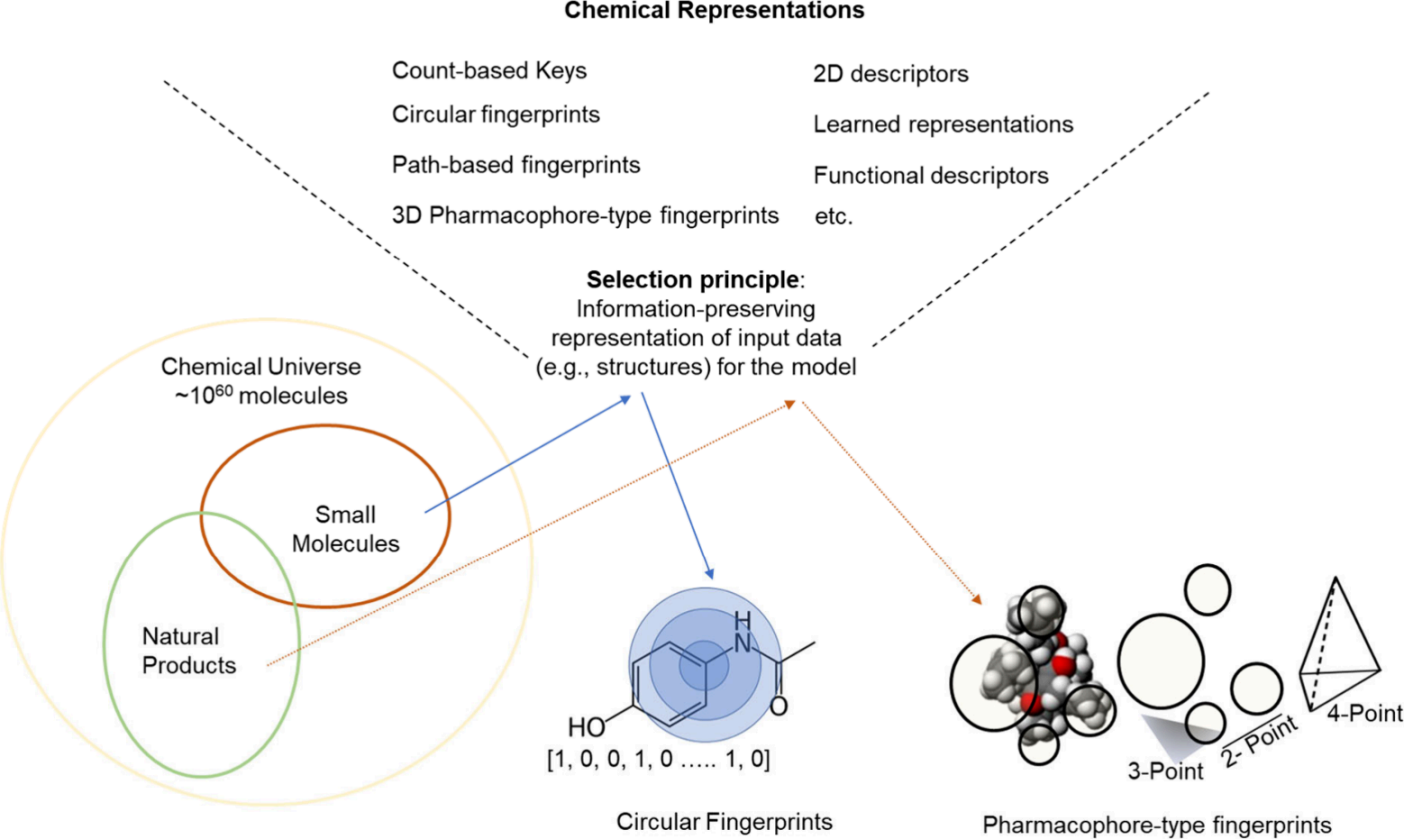
An example selection process for chemical representations. Chemical representations vary in their ability to capture the complexity of the molecular structures. When building models for specific categories, like small molecules or natural products, it is important to choose a suitable representation, based on factors such as predictive performance. For instance, circular fingerprints often work well for small molecules, while pharmacophore-based fingerprints may be better suited for natural products. However, studies show that no single representation consistently outperforms others across all QSAR data sets.^151,152^

ML models for molecular toxicity prediction that use chemical structures as their input are based on the fact that a molecule’s properties depend on its chemical structure^142,143^ and that, generally, molecules with ‘similar’ chemical structures (which can be defined in a wide variety of ways) tend to exhibit similar properties. In classical ML applied to these tasks, quantitative structure–activity–property relationship (QSAR/QSPR) models are trained on predefined feature spaces representing chemical structures (Table 3) and used to predict toxicity labels. As such, these models can only learn relationships captured by the feature representation chosen by the ML practitioner; the choice of representation strongly influences the ability to predict particular molecular toxicities (Figure 3 and described in more detail in subsequent sections).^1,144^ Descriptors derived from a molecule’s structure are used to represent its chemical and physical properties in this particular feature space, and hence model performance (and interpretability) is intrinsically linked to feature choice. For example, physicochemical properties of a structure are related to ADME end points such as permeability or solubility; omitting features such as charge or molecular weight will generally decrease model performance for those end points. Alternatively, features can be learned directly from chemical structures, such as structural keys, chemical fingerprints, etc., but each featurization method is based on inherent assumptions that might not apply in the universal chemical space, encompassing approximately 10^60^ compounds.^36,145^ Further, structure–property dependencies are often not smoothly related: exceptions to the general structure–property rule are called activity cliffs—structurally similar compounds that exhibit significantly different properties.^89^ Activity cliffs test the limits of structure-only prediction and require experiment-based inputs for successful prediction, whether biological or physicochemical assays differentiate structurally similar compounds. Overall, the choice of features to represent a molecule is nontrivial.

**Table 3:** Most Commonly Used Representations in ML Models, Such as Those Used to Predict Compound Toxicity

Representation	Description	Examples	Advantages	Limitations	Implications for ML Model Performance
Structural keys	Bit format indicating the presence/absence of specific substructures; was intended for cataloging compounds and substructure filtering	MACCS keys^155^	High interpretability; often direct correspondence to molecular substructures	Limited chemical space coverage; no 3D information present; missing information on some substructures	Limited coverage of chemical space, because it was not originally intended to fully represent structure (but empirically often performing rather well)
Chemical 2D Fingerprints	Encodes chemical structures into a fixed-size bit string, capturing molecular connectivity	Extended Connectivity Fingerprints (ECFPs)^161^	Compresses feature space; captures broader and more specific chemical space than fixed substructure keys	No 3D information present; algorithms are predefined to generate fingerprints from connectivity	Balances coverage and compactness. It is often possible to obtain structural contributions to model predictions. Often favored in benchmarks, partially due to data set biases (especially analogue bias)
2D Descriptors	Properties calculated from simple two-dimensional (2D) chemical structures	Hydrophobicity, E-state indices, polar surface area, molecular weight and other physicochemical properties^143^	Provides insight into molecular properties; interpretable because related to measurables	Does not capture spatially dependent information of molecular structure	Enhances model interpretability and predictive power (for example, logD is often predictive of complex toxicity)
3D Descriptors	Properties calculated from the three-dimensional structure of molecules, considering spatial arrangements and capturing stereochemistry	Solvent accessible surface area with positive partial charge in the structure,^166^ Weighted Holistic Invariant Molecular Descriptors (WHIMs) and Grid-Independent Descriptors (GRINDs)	Captures detailed structural information; potential to encode other 3D properties such as surface area	Computationally intensive due to conformational sampling and complex encodings; usually, bioactive conformations are unknown	Improves model precision by leveraging detailed 3D structural information, essential for tasks requiring stereochemical details (but has conformational sampling trade-off)
2D graph-based representations^153^	Encode the two-dimensional connectivity of molecules	Message-Passing Neural Networks (MPNNs), graph transformers	Alternative to 2-dimensional fingerprints and leverages deep learning architectures	Computationally efficient compared to 3D models, but lack spatial information	Useful for predicting molecular properties related to connectivity
3D graph-based representations^153^	Encode the three-dimensional structure of molecules, considering spatial arrangements	Message-Passing Neural Networks (MPNNs), graph transformers	Captures stereochemistry and has potential to encode other properties such as surface area	Computationally intensive and increased memory requirements	Potential to improve model precision by leveraging connectivity from structural information
Descriptors from experiments	Experimental data that capture changes in a biological system upon perturbation with chemicals	Cell Painting, Transcriptomics, Proteomics, etc.	Captures biological *response* space that may not be captured by structure-derived representations	Requires experiments with actual synthesized compounds, processing, quality control, etc. to prepare feature space	Potential to improve the applicability domain of models and identify activity cliffs, as well as interpretability in cases where features are found to be predictive for end point of interest
Learned/pretrained representations	Automatically extract features from input data (whether chemical structures or experimental data) using pretrained encoders (supervised/semisupervised) and convert them to a latent space	ChemProp, CLOOME	Captures complex patterns and relationships; adaptable to various data types; improves with more data	May require large data sets for training and generation of features that are suitable for future use cases; can be less interpretable than traditional descriptors	Enhances model accuracy and generalizability; potential to integrate diverse data sources for a comprehensive understanding of molecular behavior

Besides chemical structure-based descriptors, phenotypic data are sometimes available to aid predictions. These omics descriptors can be collected from cells treated with each chemical, including readouts like the Cell Painting assay, transcriptomics, proteomics and metabolomics.^5,146−148^ These are relatively unbiased or hypothesis-free descriptors, and unlike computed structural features, they report on the actual interaction of the chemical with a biological system. The experimental costs for phenotypic assays can be high, but descriptors from them have been shown to improve the chemical applicability domain of models to predict toxicity.^146,149,150^ Nevertheless, representations from phenotypic data sets might not always be relevant to predict a specific biological end point; that is, they have limitations in their biological applicability domain (i.e., mode of action coverage). The signal present in phenotypic data needs to be established and validated for each end point of interest, in many cases empirically. Whereas Pillar 1 covered compound standardization, the focus of this Pillar is on representations directly derived from chemical structures, given that this representation does not require experimentally determining compound effects, and it is hence universally applicable.

## Molecular Keys and Fingerprints

**Figure 4:**
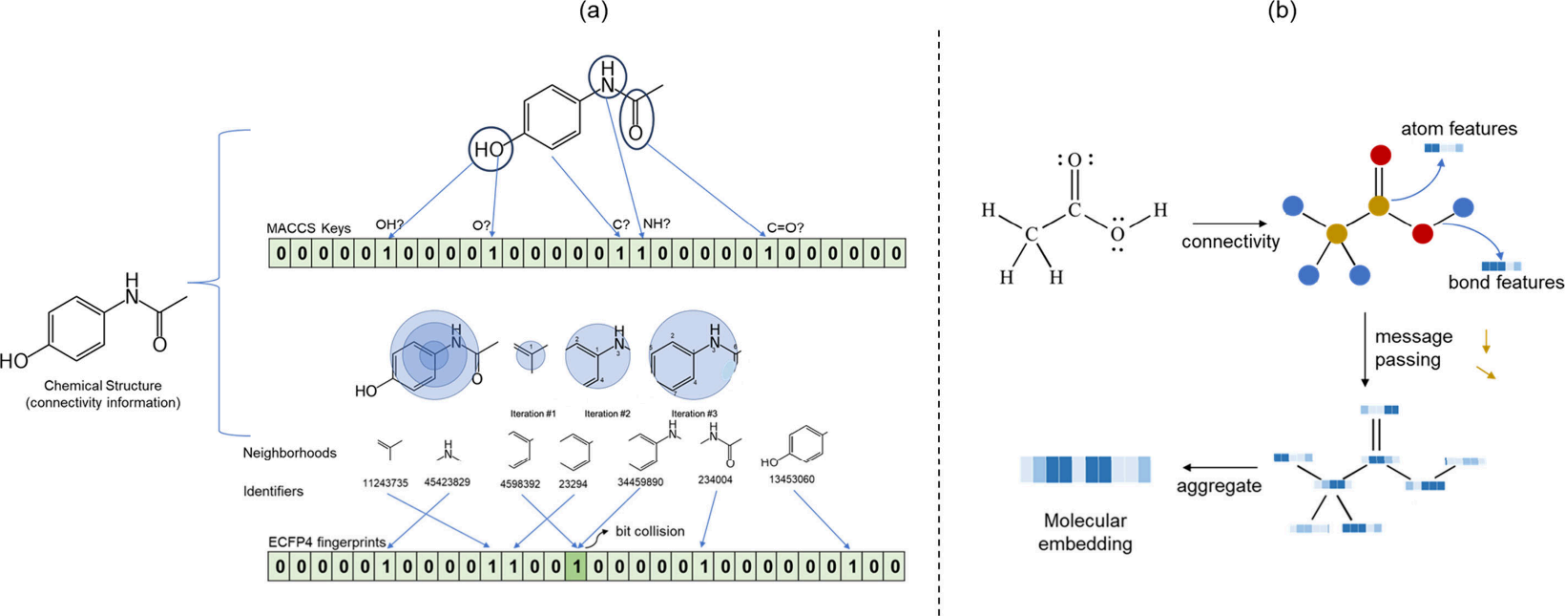
(a) Encoding chemical structures using Molecular ACCess System (MACCS) keys, top, and Extended Connectivity Fingerprints (ECFP), bottom. MACCS keys, originally used for cataloging, directly map specific predefined features with bits representing distinct rules, such as the presence of atoms, bonds, etc. ECFP encodes a broader range of substructures. (b) Learned representations encapsulate the learned embeddings of atoms within a molecule aggregated to form a comprehensive molecular embedding. Typically, they focus on molecular topology and connectivity with nodes as atoms and edges as bonds with an iterative message-passing process.

One common representation of molecules using only their chemical structure as input is molecular keys (Figure 4a, top).^153^ Structural keys such as Molecular ACCess System (MACCS) keys are a straightforward representation, originally developed for cataloging compounds, but also performing surprisingly well in predictive models.^154^ These keys encode molecular structures into a 166-bit format (publicly available) or a 960-bit format (in the commercial implementation) by recording the presence or absence of specific predefined substructures or chemical patterns within a molecule, such as the presence of ring systems, particular functional groups, etc.^155^ Because each bit in structural keys corresponds to a predefined feature, often a molecular substructure, it is easy to interpret what features are vital for the model’s predictions. Thus, encoding molecules using structural keys facilitates interpretation due to the direct correspondence between specific values in the fingerprint and particular predefined features.^156,157^ However, these predefined features limit the chemical space represented, because structural keys were not intended to capture all relevant aspects of the chemical structure.

An alternative to structural keys are chemical fingerprints, such as extended connectivity fingerprints (ECFPs),^158^ which represent the chemical structure of a molecule as a count-based vector or fixed-length binary vector (Figure 4a, bottom). Count-based fingerprints in principle benefit by counting the presence of multiple substructures, which can be especially useful in the case of, for example, repeat units (peptide backbones, terpenes, sugar attachments, etc.). Although count-based fingerprints capture more information, not all ML methods can leverage them as they are always very large (∼2^32^-1).^159^ Thus, counts are usually converted into binary bits, and therefore some details about the molecule’s specific structural or chemical features are lost. This loss of information does not necessarily mean lower predictive power as the signal and predictive ability will be retained if the relevant information is retained. As for interpretability, it is possible to store a correspondence table between bits and chemical substructures to allow the unambiguous interpretation of the bits in a chemical fingerprint.^160^ Due to the vast number of possible substructures, most commonly employed chemical fingerprints use a folding and hashing algorithm to encode the chemical structure into a fixed-size bit string.^161^ Here, the same bit could correspond to multiple substructures, known as a hash collision (Figure 4a). The transformation is usually one way that introduces ambiguity, as the relationship between the original substructures and the resulting bits is not easily discernible. Overall, molecular fingerprints have been successful in toxicity prediction tasks and capture certain aspects of the chemical structures being analyzed; they are able to encode wider aspects of the chemical structure than predefined keys.

## 2D Descriptors

2D continuous descriptors are numerical properties calculated from a molecule’s 2D graph representation, typically derived from the connection table, molecular topology or chemical graphs, such as hydrophobicity, topological polar surface area^162^ and E-state indices.^163^ This level of information includes details about how atoms are connected within the molecule but does not account for the actual spatial arrangement of atoms in three-dimensional space. Physicochemical descriptors are often highly relevant to the effects of drugs within biological systems and they are readily interpretable, since they correspond to measurable properties of a molecule.^164^ Further, 2D descriptors are more computationally efficient to calculate, given that they are derived solely from molecular connectivity matrices. In summary, using 2D descriptors for molecular representation can add information to chemical structural information, such as chemical fingerprints, and enhance predictive accuracy.^165^ The quality of data, but then also problem-relevant and consistent preprocessing, is a key factor controlling model quality in cheminformatics models.

## 3D Descriptors

3D descriptors are calculated using the three-dimensional structure of a molecule, taking into account the spatial and geometric properties, including bond angles, distances, and overall geometry (Table 4). 3D descriptors can distinguish different three-dimensional conformations, but require accurate 3D structures, which are not always available or reliable, and the generation thereof leads to ‘combinatorial explosion’ (a large number of possible conformations). 3D information is needed in some cases such as for docking, where a spatial fit of the ligand into the target is performed.^167^ Studies have reported 3D descriptors to be more effective than 2D descriptors for predicting biological targets of ligands with low structural similarity, given the ability of 3D descriptors to capture pharmacophoric alignments.^168^ Overall, 3D descriptors can capture critical stereochemical and conformational information that can enhance predictions involving complex interactions and stereochemistry; however, they intrinsically need more computing resources to calculate, in particular, due to conformational sampling. When it comes to the information content that they contain, the signal-to-noise ratio needs to be considered (which does not always increase with descriptor complexity). Moreover, benchmark data sets often contain analogues that can already be well predicted by 2D descriptors, thereby intrinsically favoring 2D descriptors (and disfavoring 3D ones that are computationally expensive).

**Table 4:** Commonly Used 3D Descriptors

3D Descriptor	Description	Source
MoRSE Descriptors	3D molecular representations of structure based on electron diffraction descriptors	(173)
Gravitational Index Descriptors	Based on analogies to gravitational physics, it measures the distribution of atomic mass within a molecule	(174)
3D Autocorrelation Descriptors (3DAc)	The relative position of atoms (or atom properties) based on the separation between atom pairs in Euclidean distance	(175)
RDF (Radial Distribution Function)	Provides information about the probability distribution of interatomic distances within a molecule	(176)
WHIM Descriptors	Weighted Holistic Invariant Molecular descriptors are derived from the atomic coordinates of a molecule and capture features like size, shape, symmetry, and atom distribution	(177)
Flexophore/Pharmacophores	Uses a reduced graph with atom types and encodes molecular flexibility through node distance histograms across diverse conformers	(178)

Among the challenges when incorporating 3D descriptors into ML models, the most common is the conformation-dependent nature of most 3D descriptors; this dependence results in substantial variations of the descriptors based on the conformer selected for descriptor calculation. In principle, an ML model could leverage a set of descriptors derived from an ensemble of conformations to capture the inherent flexibility of molecules. However, typical data sets do not map activity to different conformations and 3D representations are mostly incompatible with most contemporary ML algorithms, which are designed to map a single instance to a single label. Multi-Instance Learning (MIL) offers a solution by representing each ligand as a bag containing multiple instances (conformers), and the task is to predict a property or activity associated with the entire bag rather than individual instances. MIL models are still in early stages of development and their application in toxicity prediction is yet to be validated.^169,170^

Studies combining 2D and 3D descriptors sometimes yield better performance due to their complementary information.^171^ In theory, 3D information such as stereochemistry is responsible for the selectivity in binding affinity of molecules to many protein targets.^111^ In practice, for certain bioactivity prediction tasks, 2D descriptors might outperform 3D due to a more favorable signal-to-noise ratio in descriptor space (e.g., fewer irrelevant conformations), and/or possibly the analogue bias in benchmark data sets.^152,171,172^ Thus, the choice between 2D and 3D descriptors is specific to the problem statement and data set’s chemical space and hence needs situation-specific exploration.

## Graph-Based Representations

**Figure 5:**
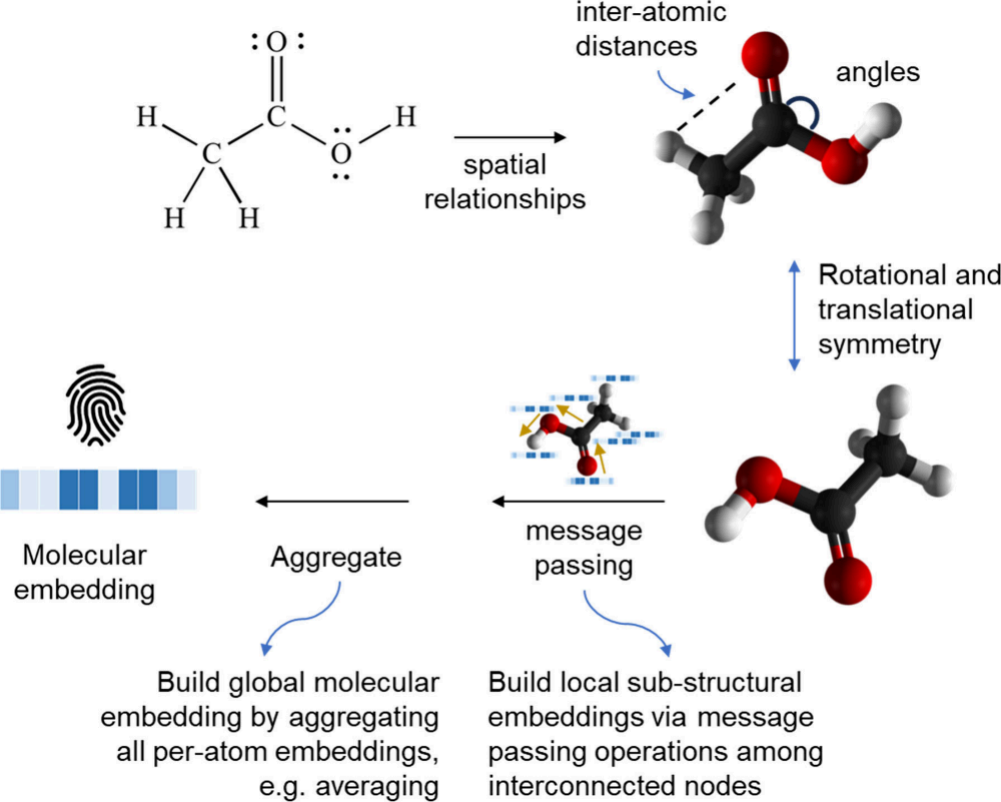
3D Graph Neural Network (GNN) architectures applied to molecular representations. 3D geometric GNN representations incorporate spatial relationships and geometric transformations to capture three-dimensional information, such as bond angles.

Graph Neural Network (GNN) architectures for molecular toxicity and activity prediction have emerged as an often more expressive alternative than using binary fingerprints with classical ML models (Figure 4b). GNNs can directly learn molecular representations from chemical structures on the fly as the model is trained, which eliminates the need for manual feature engineering and enables customizing representations to specific data sets or tasks. These representations are learned through iterative rounds of so-called ‘message passing’ (Figure 4b). Briefly, molecules are initially featurized as graphs, with atoms as nodes and bonds as edges, and atoms and edges are initially described by simple physicochemical properties. While 2D GNNs use only simple atom and bond properties to provide node and edge features, 3D GNNs (Figure 5) additionally incorporate 3D coordinates, providing spatial information for atom and edge features, and may leverage relative coordinates in their message passing rules to capture the molecule’s geometric configuration. Then, atom and bond representations are made progressively more abstract by transforming and combining the descriptors of those atoms and bonds that are neighbors in the graph. In this way, a message passing on the graph makes atoms and bonds incorporate information about their chemical environment. Finally, the representations of atoms and bonds can be combined to obtain representations for the whole molecule.^179,180^ Additional chemical- and surface-related information can also be provided as initial input features to GNNs. Alternatively, transformer architectures have also emerged as a powerful approach for graph-based modeling, with a flexible mechanism to capture long-range dependencies in molecular structures.^181,182^

The use of message passing in GNNs ensures that all operations performed by the model are independent of any particular ordering of the constituent nodes, leading to model reasoning that is purely based on the aggregated information from a node’s local neighborhood. GNNs can serve as an encoder that processes a molecular graph, with nodes representing atoms and edges representing bonds (Figure 5), to generate a compact vector capturing the chemical structural information in the context of the pretraining task.^183^ The GNN, pretrained on large chemical spaces, refines these representations to better capture features influencing target outcomes like compound toxicity. Pretraining can be supervised, using labeled data sets to predict molecular properties, or semisupervised, leveraging labeled and unlabeled data to refine latent space representations. Message-passing neural networks (e.g., Chemprop) have been proposed as encoders to generate latent space representations of molecules^180^ and used to predict various properties including toxicity (discussed later as representation learning).^184,185^ An emerging class of geometric GNN architectures for molecular toxicity prediction represents molecular graphs in three-dimensional space and additionally incorporates inductive biases that make them invariant to global rigid transformations of molecules—rotations and translations of the 3D coordinates.^186−188^

Gao et al. showed that models with access to three-dimensional structural information on the protein–ligand complex outperform approaches that use 2D fingerprint representations for the prediction of protein–ligand binding affinity.^152^ Broadly, for those situations where the binding pose is relevant for an effect, using three-dimensional binding pose-dependent properties may provide a more comprehensive representation of the compound (albeit with the need to handle conformational information). However, protein flexibility is a significant factor in scenarios where binding pockets are less defined or allosteric binding occurs; the conformational changes in the protein induced by ligand binding can significantly affect the binding mode and affinity. For example, enzymes like cytochrome P450s (CYPs) as well as transporters exhibit a high degree of flexibility, further complicating the accurate prediction of ligand–protein interactions.^189^ 3D ligand information is also being used in protein–ligand cofolding, which then predicts binding, and this is rapidly evolving with recent advances in AlphaFold^190^ and RosettaFold.^191^

In reality, three-dimensional molecules have several possible conformers, with proteins and ligands existing in multiple conformations and ensembles, and their interactions can shift these conformational equilibria. As such, a single three-dimensional graph embedding may fail to consider molecules’ dynamic behavior and flexibility, which can adopt various conformations in different environments, such as rotational freedom around single bonds. For example, the conformers of acetylcholine, such as the anti and gauche forms, demonstrate variable affinity to nicotinic and muscarinic receptor subtypes.^192^ In contrast, only the anti form is preferred for catalysis by acetylcholinesterase, an enzyme critical in the breakdown of acetylcholine and, thus, in regulating neurotransmission.^193,194^ However, flexibility is not always critical to prediction for ML models—in majority of data, if an inhibitor fits the pharmacophore and effectively binds to inhibit the target, it may be sufficient to train an ML model without considering the molecule’s flexibility. Where importance of molecules’ dynamic behavior and flexibility is needed based on the specific context and the nature of the interaction being studied, one could use higher dimensional descriptors (multidimensional QSAR^195,196^) or use multiple instance learning (MIL), learning meaningful molecular representations relevant to prediction tasks from multiple ligand conformers of three-dimensional chemical structures.^197,198^ By leveraging the diverse conformations that a molecule can adopt, MIL enhances the accuracy of toxicity predictions.^199^

Whether to use 2D or 3D geometric GNNs for molecular toxicity prediction is a question of data availability and the expressivity of representations. For tasks where the conformational state of a molecule is known to be important, such as protein–ligand interactions, 3D GNNs that incorporate protein–ligand poses from docking (providing information about the ligand’s approximate binding mode) have been shown to outperform models based on 2D fingerprints that also encode protein–ligand extended connectivity, underscoring the critical role of 3D information in accurately predicting protein–ligand binding affinity.^200−202^

The advantage of 3D information compared to compressed representations has not been completely established in practice for the prediction of toxicity end points; Cremer et al. showed that 2D graph-based models produce comparable results to a 3D equivariant graph transformer model (which leverages geometry of conformers) and 3D geometry-based graph neural networks on ToxCast and Tox21 assays.^203^ Still, the authors largely attribute the comparable results to the limited data set size in toxicity data sets; also toxicity can be driven by individual chemical substructures (e.g., reactive chemical groups, electrophilicity, etc.), where 2D descriptors capture relevant information rather well. The relative paucity of 3D protein structure data means that overfitting is common in more expressive geometric GNNs, leading to most published work in molecular toxicity prediction relying on standard 2D GNNs.^204^

## Representation Learning

Representations can be learned on a given data set and then used as a feature on another prediction task, for example, in a strategy called transfer learning. Representations that have been pretrained for this purpose are relatively novel to cheminformatics compared to chemical fingerprints. Unlike hand-engineered or experimental descriptors, representation learning automatically extracts relevant features from raw data, capturing complex patterns and relationships, and is particularly powerful when handling large data sets.^106^ By contrast, they are often not beneficial on smaller data sets.^205^ Encoders are typically pretrained on related tasks to extract relevant features from raw data into a lower-dimensional and abstract representation known as the latent space. The compressed vector space retains essential features, making it easier for the models to handle complex data. Learned representations can be used downstream as feature vector inputs to another model or can be fine-tuned for a specific task.

There are various strategies to learn representations, such as message passing neural networks, discussed in the prior section. Another approach to automatically extract relevant features from raw data involves comparing and contrasting samples (including the possibility of using 3D information).^170,206^ This method, known as contrastive learning, trains a model to distinguish between similar and dissimilar pairs of data points, typically by minimizing the distance between representations of similar pairs (positive pairs) and maximizing the distance between representations of dissimilar pairs (negative pairs). Thus, contrastive learning involves creating an embedding space where similar data points are located close to each other and dissimilar points are distant. For example, in the context of compound bioactivity data, the model might be trained to recognize that two different chemical scaffolds with similar bioactivity should have similar representations, while scaffolds with different bioactivity should have distinct representations. In addition to the strategies above, another potent method for learning representations involves using neural machine translation between two semantically equivalent but syntactically distinct molecular structure notations, such as InChI and SMILES. This technique, based on continuous and data-driven descriptors,^207^ compresses the shared substantive information from both notations into a condensed, information-rich vector representation.

One of the advantages of representation learning is its ability to integrate multiple data types, such as chemical structures, biological activities, gene expression, and phenotypic profiles.^208,209^ Liu et al. introduced the Information Alignment (InfoAlign) approach, which uses the information bottleneck method to learn enhanced molecular representations by integrating chemical structure data with cell morphology and gene expression data.^209^ On the Biogen ADME data set, InfoAlign was tested across five end points: MDR1-MDCK efflux ratio (ER), solubility at pH 6.8, rat liver microsomal intrinsic clearance, human plasma protein binding (hPPB) percent unbound, and rat plasma protein binding (rPPB) percent unbound. InfoAlign outperformed traditional chemical fingerprints and other contrastive learning methods like CLOOME^210^ and InfoCORE^211^ reducing mean average errors by 6.33%, and thus learning better representations of molecules by integrating biological data.^5^ Overall, representation learning allows the integration of various task-relevant biological data in encoding chemical space.

## Feature Selection or Reduction

Feature selection or reduction is a key step in data analysis and modeling, serving as a way to reduce model variance (via the elimination of noisy features^212^) or bias (via the inclusion of relevant features).^213^ Thus, feature selection methods can enhance the signal-to-noise ratio and enable the model to concentrate on the most significant predictors. An effective and efficient feature selection method considers both feature relevance (high information content) and redundancy (correlations between features).^214^ Including irrelevant or redundant features can add noise (albeit models like partial least-squares regression, as one of few exceptions, can still work with highly redundant features), causing the model to overfit by learning the noise rather than the true underlying patterns resulting in poor generalization of new data and decreased model performance.^214^ However, eliminating useful features during the selection process degrades performance by reducing the model’s ability to capture important patterns or relationships in the data. The difficulty in practice is that data sets are limited in size, and hence feature relevance can only be assessed on the given data; relevance of features might change when new data becomes available.

Feature selection or reduction (as well as any optimization of model hyperparameters) should be done on the training data only, while the test set, on which future performance of the model is estimated, should not be used for feature selection; this would represent information leakage.^215,216^ Feature selection methods can evaluate features based on statistical criteria without involving a predictive model (‘filters’), use a machine learning model to iteratively select the best subset of features by testing performance (‘wrappers’), or be embedded within the learning process of a predictive model (‘embedded methods’).^217^

Several methods address the feature selection step (Table 5).^160^ For instance, Minimum Redundancy Maximum Relevance (MRMR) aims to select features that are highly correlated with the target variable while being minimally redundant with each other.^218^ Recursive Feature Elimination (RFE) iteratively removes less significant features based on model performance, providing an optimal subset of features and integrating well with different model types.^219^ Other techniques like Mutual Information measure the ‘information gain’ provided by each feature (that is, information provided about the target variable), capturing nonlinear relationships and aiding in feature ranking.^220^ Boruta is an all-relevant feature selection method: that is, selecting all features with a meaningful relationship to the target, whether redundant or unique. Boruta iteratively compares the importance of actual features with that of randomly shuffled features to determine significance, making it robust to overfitting and easy to interpret.^221^ Context-dependent sparse feature selection methods, such as LASSO and other L1 methods, help remove low-relevance features, which minimizes overfitting and improves model interpretability and predictive power.^222^ Principal Component Analysis (PCA) is often employed for feature (dimensionality) reduction by selecting a finite number of components that explain the majority of the *variance*, but it does *not* explicitly select features based on their relevance to a specific task, such as predicting a target variable such as toxicity. While some have proposed using PCA for feature selection,^223^ it is usually not ideal for feature selection where task-specific relevance is key.^224^ Overall, in ML models, feature selection or reduction can improve the signal in the data by removing irrelevant or redundant features, thereby enhancing model performance as well as simplifying model interpretation due to the reduced number of features (provided that suitable features exist in the first place).

**Table 5:** Table of Commonly Used Methods for Feature Selection (from the set of original features) and Reduction (new features)

Method	Description	Strategy for Selection/Reduction	Advantages	Disadvantages	Category	Reference
Minimum Redundancy Maximum Relevance (MRMR)	Selects features highly correlated with the target but minimally redundant with each other	Relation to output space	Balances relevance and redundancy, suitable for high-dimensional data	May struggle with nonlinear relationships, computationally expensive for large data sets	Supervised, Filter	(218)
Recursive Feature Elimination (RFE)	Iteratively removes features by training models and evaluating their performance	Relation to output space	Provides an optimal subset of features, integrates well with different model types	Computationally expensive for large data sets	Supervised, Wrapper	(219)
Mutual Information	Measures the information gained about one variable through the other, useful for feature ranking	Relation to output space	Nonparametric, can capture nonlinear relationships	Computationally expensive, sensitive to noise in the data, can be inaccurate as well, because the true MI often involves a very high-dimensional integral^225^	Supervised, Filter	(220)
Boruta	Iteratively compares feature importance with that of randomly shuffled features to determine significance	Variance in input space and relation to output space	Robust to overfitting, easy to interpret	Computationally expensive, may select more features than necessary	Supervised, Wrapper	(221)
Least Absolute Shrinkage and Selection Operator (LASSO)	Applies L1 regularization to linear regression to penalize coefficients, forcing some to zero	Relation to output space	Simple to implement, handles high-dimensional data well, and reduces overfitting	May select too few features if high correlation exists among predictors; difficult to interpret because it is an emergent property of the model fitting and not based on a metric calculated per-feature	Supervised, Embedded	(222)
Elastic Net	Combines L1 (LASSO) and L2 (ridge) regularization to select relevant features and handle correlated predictors	Relation to output space	Handles correlated features well, useful for data sets with more predictors than observations	Can be difficult to tune the mixing parameter between L1 and L2 regularization	Supervised, Embedded	(226)
ReliefF	Estimates feature importance by repeatedly sampling instances and comparing nearest neighbors	Variance in input space and relation to output space	Captures interactions between features, works well with noisy data sets	Sensitive to the choice of nearest neighbors, may not perform well with imbalanced data	Supervised, Filter	(227)
Genetic Algorithms	Evolves a population of potential solutions (feature subsets) using genetic operators like mutation and crossover	Natural selection and relation to output space	Flexible and adaptable, can escape local optima in feature selection	Computationally expensive, requires careful tuning of parameters like mutation rate	Unsupervised, Embedded	(228)
Principal Component Analysis (feature reduction method)	Dimensionality reduction technique that projects data onto orthogonal axes to maximize variance. Lower-ranking features can be discarded.	Variance in input space	Reduces dimensionality, improves computational efficiency, and removes multicollinearity	Information loss occurs, and the principal components often lack direct interpretability	Unsupervised, Filter	(223)

## Pillar 3: The Choice of Model Algorithm

After choosing a suitable representation of chemicals, the choice of modeling algorithm is the next critical factor determining the performance and usefulness of an ML system for molecular toxicity prediction. In the context of ML, a model is a mathematical system designed to make predictions based on input data. Models use a set of features (as outlined in the previous section) to capture the essential characteristics of the molecules under study.^143^ Most models make predictions for a data point (chemical) by using that data point’s features and a set of model parameters that need to be fitted (trained) to the data. In a simple linear model, these parameters determine the weight of each descriptor in making a prediction; however, in more complex models, those relationships can be more difficult to interpret. Fitting a model involves adjusting these parameters to minimize the error between the model’s predictions and observed outcomes from the training data. For a review on classical ML methods, the reader is referred to Mitchell^229^ and Lavecchia et al.^230^

## Bias-Variance Trade-Offs

**Figure 6:**
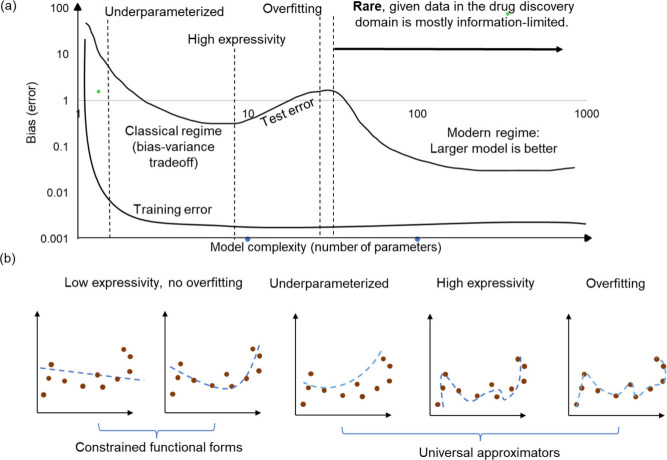
(a) Theoretical depiction of how increased model complexity reduces bias but increases variance, highlighting the tipping point where overfitting begins. Yet, recent research reveals a “double descent” regime (toward the right), as described in the main text. (b) Data points (in brown) alongside fitted models (blue dashed lines) to visualize the difference in the fit quality across various model complexities, from simple models with constrained functional forms to neural networks that are universal approximators with high expressivity but prone to overfitting.

As with selecting a feature representation, the choice of ML models also presents a trade-off between having lower prediction power (or poor ability to generalize to novel chemical space) and overfitting (the ability to fit the training set well, but at the cost of being less able to generalize to unseen structures). Usually, ML models with constrained functional forms can only approximate a limited set of target functions, limiting their expressivity but preventing overfitting, which hence represents a trade-off (Figure 6). For example, linear regression models assume a linear relationship between the input features and labels, which limits the expressivity of the model to linear functions. Thus, linear regression models can be insufficient for capturing the complexity and variability present in domains where nonlinear patterns are common but can be a useful choice when data is scarce and have the advantage of being readily interpretable. On the other hand, deep learning models, such as multilayer feed-forward neural networks are more flexible (universal) approximators and can capture complex nonlinear functions but require sufficient size, data, training, and hyperparameter tuning (network architecture, etc.), and come at a cost of interpretability.^231^

The trade-off between poor predictive power and overfitting is a fundamental principle of ML known as the bias-variance trade-off (Figure 6).^232^ Both bias and variance relate to the model’s ability to learn feature-target relationships from the training data set. Bias refers to the model’s tendency to not capture (and hence overgeneralize) real feature-target relationships that are present in the training set. Bias can be high when a simple model is used, such as a linear model or a small neural network, or when the model or training algorithm includes assumptions about the shape and nature of the data that may not be true for the data seen during deployment. Variance, on the other hand, refers to the model’s tendency to capture spurious relationships in the training data set (such as noise). For example, the presence of a rare functional group in a single toxic compound (where this functional group, however, is not related to toxicity) might lead the model to incorrectly predict that other compounds with this functional group are toxic. Overly simplistic models exhibit high bias and fail to learn from the data adequately but are less likely to overfit to noise or spurious correlations in the training data and can have the ability to extrapolate better to new data. Due to their simplicity, interpretability, and robustness to noise, classical models, such as linear models (Table 6), have been widely used with binary fingerprints to predict toxicity.^1,233^ In contrast, complex models can model intricate relationships but are more likely to learn spurious correlations and overfit.^234^

**Table 6:** Evaluation of Commonly Implemented ML Algorithms Concerning Their Simplicity, Interpretability, Robustness to Noise, Functional Form, Expressivity, and Bias-Variance Trade-Off When Used in Toxicity Prediction

ML Model Type	Complexity and Interpretability	Robustness to Noise	Functional Forms Approximated	Expressivity (ability capture complexity, variability of data)	Bias-Variance Trade-Off
Linear Models	Low complexity; high interpretability	Low	Linear relationships	Low	High bias, low variance—good for simple relationships but may underfit complex data
*k*-Nearest Neighbors (kNNs)	Low complexity; high interpretability^238^	Low	Local properties; distance-based	Depends on the input data; usually high	Low bias, high variance (with the small *k*-values that are typically necessary in moderate data regimes)
Decision Trees (DTs)	Moderate complexity; high interpretability	Moderate	Hierarchical decision rules	Moderate	Can vary; e.g., deep trees have low bias but high variance
Random Forests (ensembles of DTs)	High complexity; moderate interpretability	High	Ensemble of decision rules	High	Reduced variance through averaging, better generalization, and flexible control of bias and variance, but can overfit if not properly tuned
Gradient Boosting Machines (GBMs, typically ensembles of DTs)	High complexity; moderate interpretability	High	Ensemble of decision rules	Very high	Flexible control of bias and variance but can overfit if not properly tuned
Support Vector Machines (SVMs)	Moderate complexity; low interpretability	High	Linear and nonlinear (with kernels)	High	Good balance, but the choice of kernel affects the complexity
Neural Networks (NNs, multilayer perceptron/feedforward)	Complexity and interpretability depend on architecture (Shallow NNs have single hidden layers); while Deep NNs have multiple hidden layers, and hence higher complexity and lower interpretability than classical models	Depends on architecture (dropout, regularizers)	Virtually any functional form	Very high	Flexible control of bias, high variance—powerful but prone to overfitting (requires regularization), requires large data sets
Graph Neural Networks (GNNs)	High complexity; low-to-moderate interpretability	High	Complex graph structures	Very high	Balances bias and variance through structure-aware processing; can be prone to overfitting with highly expressive models, requires large data sets

Recent deep learning research has challenged and modified the traditional understanding of the bias-variance trade-off by introducing the concept of double descent (Figure 6a).^232,235^ When a model becomes too complex, it initially leads to overfitting and high variance (Figure 6b). As the number of parameters approaches the number of observations, small changes in the data cause large changes in the model. However, as complexity is increased further, the error surprisingly decreases again, an effect known as ‘double descent’.^235^ At some point, highly overparametrized models can hence often generalize well, despite having at the same time a large number of parameters. Double descent challenges the traditional U-shaped curve of bias-variance trade-off, revealing a more nuanced relationship between model complexity, training time and data set size with the generalization error.^236^ Nevertheless, its relevance to toxicology data, especially in low-data scenarios, may at the current point in time be limited. There are only a few instances where this effect has been observed in the drug discovery domain. For example, when training NeuralDock to predict the binding energy and affinity of a protein-small molecule pair based on protein pocket 3D structure and small molecule topology, the authors observed the second descent because the 46 million parameters was much higher than the roughly 2000 data points used in training; the authors noted that the second descent contributed to its high accuracy.^237^ However, in smaller data sets typically used in ML models for compound toxicity, encountering double descent is unlikely.

Algorithms described in the prior section (Table 6) are widely employed in molecular and toxicity prediction (Table 7), and are based on features like molecular structure, chemical properties, and biological data. For further reading, we recommend the compendium of articles in “Introduction to the Special Issue: AI Meets Toxicology” and “Analysis of Tox24 challenge results”, which offers a comprehensive exploration of AI/ML applications in toxicology, presenting advanced methodologies and diverse case studies that showcase the full potential of these technologies.^239,240^

**Table 7:** Reviews of the Predictive Model Algorithms Used in Predicting Toxicity

Review	Year	Algorithms Described/Discussed	Reference
Mitchell et al.	2014	Discusses common supervised learning algorithms: Artificial Neural Networks (ANNs), Random Forest (RF), Support Vector Machines (SVMs), *k*-Nearest Neighbors (kNNs), and Naïve Bayes (NB) classifiers.	(229)
Ekins	2014	Discusses Bayesian models, SVM, kNN, and RF for predicting various toxicities, such as hepatotoxicity and cardiotoxicity.	(241)
Lavecchia et al.	2015	Covers algorithms like SVM, Decision Trees (DT), RF, NB classifiers, kNN, and ANN.	(230)
Raies and Bajic	2016	Discusses rule-based systems, structural alerts, read-across methods, dose–response models, pharmacokinetic/pharmacodynamic models, and QSAR models.	(242)
Baskin	2018	Explains methods like multiple linear regression, kNN, SVM, DT, RF, and deep learning, along with unsupervised methods like Kohonen’s self-organizing maps.	(243)
Yang et al.	2018	Describes ML approaches for predicting chemical toxicity, including SVM, RF, deep learning, and structural alerts for toxic substructure identification.	(244)
Vamathevan et al.	2019	Discusses ML applications in drug discovery, highlighting target identification, clinical trials, and challenges like data quality and model interpretability to reduce clinical failure rates.	(245)
Ciallella et al.	2019	Reviews AI applications in computational toxicology, covering data-driven and mechanism-driven models such as Adverse Outcome Pathways using public data sets and high-throughput screening.	(246)
Jiménez-Luna et al.	2020	Focuses on deep learning, explainable AI (XAI) techniques like feature attribution, gradient-based methods, surrogate models, and instance-based approaches for drug discovery model interpretability.	(247)
Wang et al.	2021	Covers regression models, kNN, DT, NB, SVM, RF, ensemble learning, ANN, Deep Neural Networks (DNNs), and CNN for modeling.	(248)
Dara et al.	2022	Describes methods such as SVM, RF, Multi-Layer Perceptron (MLP), deep learning, Autoencoders, and Reinforcement Learning in drug discovery.	(249)
Cavasotto et al.	2022	Reviews recent ML advances in toxicity prediction, noting challenges, methods, and relevant databases.	(57)
Tran et al.	2023	Focuses on key toxicity properties (e.g., hERG inhibition, drug-induced liver injury) and ML models like RF, SVM, and DNN in toxicity prediction.	(250)
Guo et al.	2023	Covers ML and deep learning models like SVM, RF, kNN, ensemble learning, MLP, Convolutional Neural Networks (CNNs), and Graph Convolutional Networks (GCNs) for toxicity prediction.	(251)
Tonoyan et al.	2024	Highlights opportunities in supervised, unsupervised, and reinforcement learning for toxicology applications.	(252)

## Read-Across Models

While in some cases the quantitative or qualitative prediction of a compound property across all chemical space and all output property space is desired, especially in the safety area, sometimes other approaches are employed. One such approach is known as ‘read-across’, which relies on structural similarity *and contextual information* to predict the toxicity of a chemical substance.^253,254^ Read-across is particularly popular due to its application context and the legal environment, which impose distinct requirements, such as interpretability and confidence in assigning compound properties. The process involves first identifying molecules that belong to the same chemical series or category as a query compound, based on the assumption that structurally similar compounds will exhibit similar biological activities due to shared mechanisms of action. Once appropriate analogs are identified, toxicological data from these substances are “read across” (data gap filling) to the target chemical to fill data gaps and inform safety assessments. A *k*-nearest neighbor chemical structure-based strategy (often with *k* = 1) is one example of a read-across strategy. Given the regulatory environment and cultural practice in toxicology, ‘machine learning is not everything’: approaches can and should be tailored to the particular use case.

## Generative Models

Related to *predictive* models are *generative* models, which in addition to learning patterns from a training set are able to generate novel chemical structures and which can include toxicity as one factor for optimization. Unlike predictive models, where one predicts the label for a given molecule, generative models hence ‘design’ the molecule itself given the desired label.^255^ These models are typically optimized over iterations using scoring functions that predict desired properties including novelty, activity, and synthesizability in addition to toxicity.^256,257^ Recently, generative models have been used for this, including Variational Autoencoders (VAEs), Generative Adversarial Networks (GANs), and Large Language Models (LLMs).^256,258^

## Variational Autoencoders

**Figure 7:**
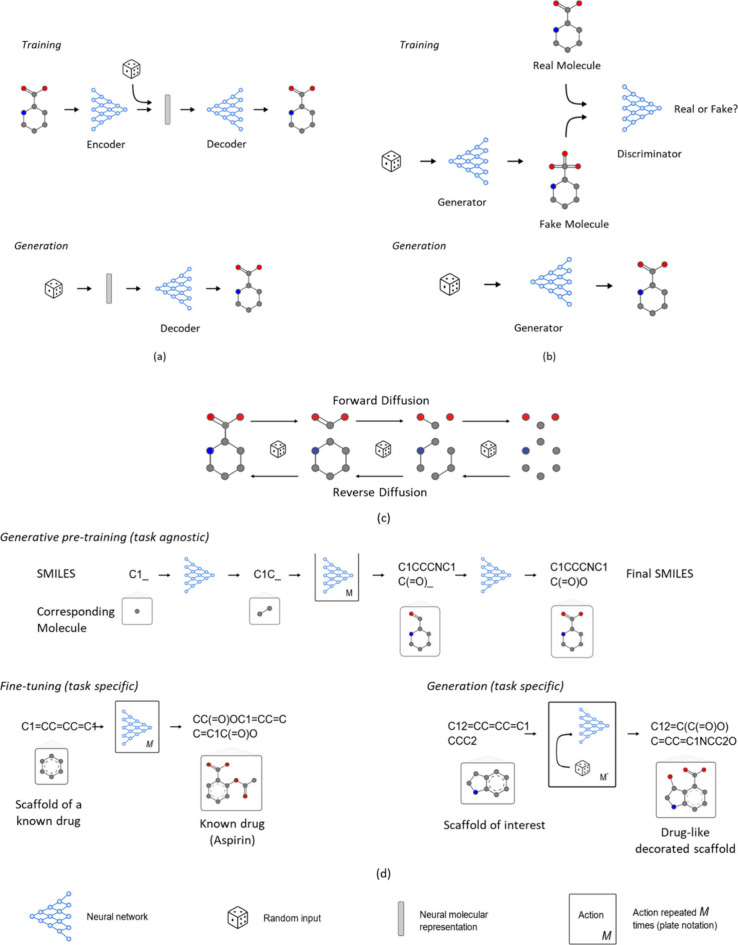
(a) Variational Autoencoders (VAEs) encode molecules into a latent space representation and then decode to generate new molecules, aiding in the exploration of chemical space. (b) A conceptual framework behind Generative Adversarial Networks (GANs), illustrating how a generator creates new molecule designs and a discriminator evaluates their realism, facilitating the generation of novel compounds. (c) Diffusion Models, a class of generative models that learn to generate data by transforming noise. (d) Large Language Models (LLMs) are also used to design molecules, emphasizing their ability to predict molecular properties and generate new compounds based on learned patterns.

Variational autoencoders (VAEs) are a class of generative models that learn representations through compression and reconstruction (Figure 7a).^259^ In a classical autoencoder, an encoder network maps data points from a high-dimensional input space (e.g., 2D or 3D molecular structure space) to a lower-dimensional latent space. Then, a decoder network is reconstructed back to the original input space, attempting to recover the data points that were fed into the encoder network. In contrast, in a VAE, the encoder maps a point from the input space not to a specific point in the latent space but to the parameters of the Gaussian distribution in the latent space, and the decoder tries to restore the point in the input space based on the result of sampling the Gaussian distribution. The presence of the sampling stage makes the VAE a generative model. Since reconstruction is required, but at the same time the latent space is smaller than the input space, the VAE encoder network must learn a compressed representation of the data that captures its most important features and ignores irrelevant ones. Because the ideal distribution in the trained latent space is known, novel molecules can be generated with the trained model by sampling points in the latent space and passing them through the decoder.

VAEs are popular general models for molecular representation learning and generation.^260,261^ Molecular generation with VAEs can be biased to optimize various objective functions such as docking scores^262,263^ and drug-likeness.^264,265^ In the context of toxicity prediction, conditional VAEs^265^ have been applied to explore low-toxicity regions of chemical space, using a scalarized objective that combines labels related to different forms of toxicity (cardiotoxicity, mutagenicity, pulmonary toxicity and skin sensitivity).^255^ Other studies have used arithmetic in the latent space to predict polypharmacology, adding or subtracting latent representations corresponding to treatment with different substances in order to simulate cellular states (as described by cell morphology and gene expression) when subjected to multiple compounds.^266^

## Generative Adversarial Networks

Generative Adversarial Networks (GANs) are a class of generative models consisting of a generator and a discriminator, two neural networks, that are trained simultaneously through adversarial training.^267^ A conceptual framework behind a GAN is depicted in Figure 7b. The generator network in Figure 7b is trained to create novel molecular structures, while the discriminator network evaluates whether these structures are realistic. At each training iteration, the discriminator is shown a batch of molecules that either were sampled randomly from a set of real molecules (“real”) or were artificially generated by the generator (“fake”). Using these examples, the discriminator must learn to determine whether a molecule is real or fake. In turn, the generator must learn to fool the discriminator by proposing molecules that mimic those in the real set. We can bias the generator to suggest compounds with certain properties, for example low toxicity, by designing the set of real molecules so that it features those properties.

Recent examples of works that explore the application of GANs to toxicity in medicinal chemistry are MedGAN, ToxGan and TransOrGAN. MedGAN was developed to generate new quinoline scaffold molecules from complex molecular graphs.^268^ The best model included in this study generated 25% valid compounds, of which 92% were quinolines of up to 50 atoms. Around 22–31% of the generated molecules were predicted to be nontoxic across the 12 Tox21 end points.^269^ That being said, the generation of quinolines is a task also easily achievable by a medicinal chemist, and the impact of such models in practice remains to be seen. In another study, ToxGan was used to predict new animal study results from historical data.^270^ Instead of training on molecular structures, the authors used repeated dose transcriptomic profiles from *in vivo* rat studies (Open TG-GATES) to train a GAN that generated transcriptomic profiles for compounds of interest. They could generate highly similar transcriptomic profiles that showed over 87% agreement in Gene Ontology with the actual gene expression data. Finally, TransOrGAN allowed molecular mapping of gene expression profiles based on rat RNA-seq data from 288 samples in nine different organs of both sexes and four developmental stages.^271^ By inferring transcriptomic profiles between any two of the nine organs studied, TransOrGAN achieved an average cosine similarity of 0.984 between synthetic transcriptomic profiles and the corresponding real profiles.

The success of frameworks like MedGAN, ToxGan, and TransOrGAN demonstrates the principle of GANs in molecular design, offering a promising avenue for generating novel, drug-like compounds taking all available data into account in an unbiased fashion. As with other ML models, however, there are some ethical concerns with generative AI, as designing molecules with the reverse loss function has been shown to rediscover potent chemical toxicants.^272^

## Diffusion Models

Diffusion models have gained significant interest in drug discovery recently.^273^ They are a class of generative models that learn to generate data by transforming noise.^274,275^ Rather than training a generator to fool a discriminator, diffusion models learn to transform noise to data by learning to reverse a diffusion process that corrupts the data. The diffusion process, which is chosen by the ML practitioner and is not trained, starts with a point from the data set and progressively corrupts it to create a sequence of increasingly noisy iterates (Figure 7c). The process is chosen such that the final iterate is completely noise, i.e., has no information from the original data remaining such that it does not depend on the initial data point and has a distribution that can be easily sampled from. A neural network is then trained to reverse the diffusion process by predicting the previous (less noisy) iteration from any of the noisy iterates. This neural network is used to generate data by starting with a sample from the noise distribution and progressively predicting each previous, less-noisy iteration until it is left with an approximate sample from the original data distribution.

Diffusion models are used for molecular machine learning in two main ways. First, a 2D molecular structure consisting of atom and bond types can be generated.^276^ Second, given a pre-existing 2D molecular graph, the diffusion model generates 3D atom coordinates. For example, 3D structures can be generated for either low-energy conformers of the molecule,^277^ or for other molecular arrangements such as a ligand’s binding pose in a protein binding pocket.^278^ These two approaches can also be combined to generate both 2D molecular graph and 3D structure at once.^279^ In each case, the diffusion process (to corrupt the data) and the neural network (to reconstruct the data) must be designed to suit the structure of the data, whether they are discrete atoms and bonds or continuously varying atom coordinates.

Another important use of diffusion models is to avoid off-target toxicity by designing ligands that selectively bind to targets over specific off-targets,^280^ although it remains to be seen that such model improvement also translates to future applications (e.g., novel chemical space). Overall, diffusion models hold promise for predicting and understanding molecule binding and biological pathways *in silico*; however, care needs to be taken to properly evaluate models in a prospective setting.

## Large Language Models

A key trend in machine learning, especially with the rise of deep learning architectures, has been the scaling of expressive power and representation learning by harnessing available large-scale data sets. While traditional supervised learning relies on labeled data, which can be limited and costly to obtain, self-supervised learning enables models to learn from unlabeled data through carefully designed pretext tasks. These pretext tasks allow models to extract meaningful representations from vast amounts of real-world data regardless of annotation availability, which allows labeled data to be preserved for a later training stage where desired. This approach has been particularly transformative in language modeling, where popular models such as ChatGPT generate natural language sequences by effectively using unannotated text. Autoregressive models, such as Recurrent Neural Networks (RNNs), have also been applied to generate molecular representations like SMILES strings or molecular fingerprints (Figure 7c). These models work by predicting the next token in a sequence based on the previous tokens, making them well-suited for tasks where the structure of the molecule is represented as a sequence. When trained on large data sets of molecular sequences, these models can learn the underlying patterns and relationships that define valid chemical structures, allowing the generation of novel molecules with similar properties.

Building on the foundation of autoregressive models, the rise of Large Language Models (LLMs) for Natural Language Processing (NLP)^281−283^ has prompted applying similar architectures to molecular tasks given established conventions of treating molecular structures as sequences, especially for the purposes of generation and representation learning. LLMs are first pretrained on a large corpus of molecular data to learn general representations, which can then be fine-tuned for specific downstream tasks, such as molecular property prediction or reaction prediction. This two-step process—pretraining and fine-tuning—enables LLMs to generalize across different molecular tasks, making them a powerful tool for drug discovery and cheminformatics. LLMs for chemistry are sometimes called Chemical Language Models (CLMs). Many chemical databases describe and store molecules in sequence representations such as SMILES. This has facilitated the development of generative models that treat molecules as sequences,^260,261,284^ some of which have achieved considerable success in academia and industry.^285^

The idea of pretraining and fine-tuning in earlier QSAR models (before LLMs were used in chemistry) was inspired by neurophysiology and is the basis of the ASNN method..^286^ Fine-tuning approaches in QSAR (the so-called ‘Library Model’) increased accuracy of the logP model to predict logD7.4 values.^287,288^ One LLM architecture that has been particularly successful in NLP is the GPT (Generative Pretrained Transformer) architecture. The GPT model consists of a stack of neural blocks, each of which is inspired by the decoder of the transformer.^289^ This decoder block consists of a self-attention layer and a feed-forward layer, both wrapped in residual connections^290^ to avoid gradient degradation during backpropagation due to the very deep architecture. The training of GPT is performed in two stages: pretraining and fine-tuning (Figure 7c).^281^ During pretraining, the model learns to generate sequences from a training corpus of sequences one element at a time (since sequences are typically tokenized, elements are usually tokens in a token vocabulary; in NLP, tokens are common word fragments or subwords, whereas in molecular tasks, tokens may be common strings of characters in SMILES, e.g., strings of characters that correspond to common functional groups in SMILES representation). At each pretraining iteration, the model’s parameters are modified to maximize the conditional likelihood of a certain token in a sequence given all previous tokens in that sequence. In this way, pretraining is carried out in an unsupervised manner and it does not require manual annotations, which are often costly to obtain. In the second stage, fine-tuning, the models’ parameters are adapted to improve performance in downstream supervised tasks with labels. Fine-tuning to specific tasks can be done through several strategies, including full-parameter tuning, adapter layers,^291^ or Low-Rank Adaptation (LoRA).^292^ An example downstream task in NLP is sentiment analysis, where sentences are labeled with positive or negative sentiment labels. An example downstream molecular task is scaffold decoration (Figure 7d). In order to teach a chemical language model how to decorate scaffolds to obtain drug-like candidates with predicted low toxicity, the scaffolds of approved drugs may be paired with drug-like candidates. PromptSmiles is a recent CLM that specializes in scaffold decoration and fragment linking.^293^

LLMs and CLMs hold great potential for molecular generative AI and property prediction, including exploration of predicted low-toxicity chemical spaces, taking information on all available data into account. Generative pretraining strategy enables this class of models to learn from large unlabeled databases (e.g., Enamine REAL SPACE^294^), which is suitable for the domain of medicinal chemistry where collecting experimental labels from toxicity studies involving animal or human clinical trials is costly. LLMs and CLMs have shown high flexibility and performance in chemical tasks. Natural-language models are able to capture information about chemistry and an ability to assist with chemical tasks in natural language, including synthesis, both with^295^ and without^296^ fine-tuning for chemistry. Surprisingly, they have also been shown to produce molecular representations competitive with featurizations such as fingerprints, even when trained on natural language rather than chemical sequences such as SMILES.^297^ Specifically, representations from the NLP model LLaMA2^283^ achieved a mean AUC-ROC of 0.77 on the toxicity classification benchmark Tox21 that was comparable to Morgan fingerprints that achieved a mean AUC-ROC of 0.76 and representations from the CLM MoLFormer-XL, trained on 1.1 billion SMILES and comparatively small (just 12 layers),^284^ achieved a mean AUC-ROC of 0.78.^297^ This suggests that models for natural language hold potential in toxicity prediction (at least in the way benchmarking has been performed in those studies). It should be kept in mind that validation on limited data sets such as the above does not necessarily translate to future use cases (e.g., in new chemical space) due to analogue bias in data sets, etc. In addition, it is worth noting that data limitations currently (and likely in the foreseeable future) prevent the development of extremely large models for medicinal chemistry trained on molecular sequences such as SMILES, in contrast to LLMs such as GPT and LLaMA that can be trained on natural language (and hence a much larger information corpus). This is because even unlabeled databases represent a comparatively small sample of the chemical space (which is in the order of 10^60^ as opposed to around 10^10^ to 10^15^ in current databases).^298,299^

## Pillar 4: Validation of Predictive Models

While evaluation on validation sets is used to guide training in many ML models, once a model is trained on a data set, it must also be evaluated on an appropriate, clearly defined test set with the proper evaluation metrics.

**Figure 8:**
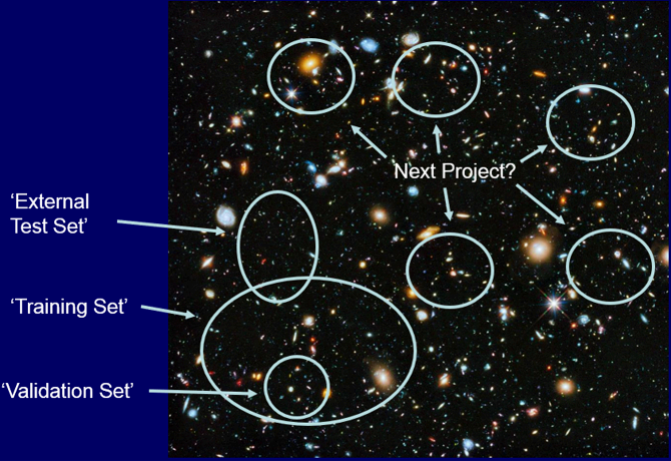
Chemical space visualized as a universe of molecules: stars represent compounds with clusters showing similar properties. The training set forms a constellation guiding predictions, while scattered stars of the test set highlight challenges in extrapolating to unseen regions, emphasizing the vastness and diversity of chemical space.

The choice of the ‘test set’ is crucial here, since one aims to extrapolate to the performance in *future* use cases from the performance obtained on the test. On the other hand, given the size of chemical space, and that in most cases properties behave differently in different areas of chemical space, this is no trivial feat, as illustrated in Figure 8 below. While evaluation of the model is performed on an ‘external test set’ (which may not be entirely external, since it is derived from an existing, split data set), future use cases are by definition in *different* areas of chemical space, and hence performance extrapolation is in practice often *not* trivial to perform. The closer to the future use case, the better the choice of the test data set in general.

We discuss two major ways to assess the models in the following: (1) retrospective validation, which holds out test compounds from the same data source, for example, by splitting the set into training and test data, and (2) prospective validation, running separate experiments on a new set of compounds.^300^ Beyond validation type, realistically assessing the performance of ML models in predictive modeling depends on selecting appropriate numerical evaluation *metrics* to understand the model’s accuracy and generalizability.^33^ The chemical space in which the model can make reliable predictions (known as the *applicability domain*(301)) should also be clearly defined following OECD principles (as discussed earlier in this work). We discuss various ways to define the applicability domain of models and common approaches to understanding models via feature importance and mechanistic analysis.

## Choosing Evaluation Metrics for Classification

ML models must use relevant performance metrics that are calculated on definitive predictions, along with confidence measures, applicability domain measures, etc.^302,303^ in order to be helpful for decision making. There are many evaluation metrics for predictive models; each emphasizes a different aspect (or a combination of aspects) of model behavior. Bender et al.^33^ provide a list of recommended metrics in the evaluation of ML models in the chemical sciences. Here, we focus on some common metrics that should be considered when predicting assay outcomes from high-thoughoutput screens, toxicity-based assays, and regression outputs.

For binary classification models, predictions are classified into two classes (e.g., positive and negative negative). A confusion matrix helps to visualize the performance of a classification model by organizing predictions into four categories:

- True Positives (TP): Correct predictions of the positive class
- True Negatives (TN): Correct predictions of the negative class
- False Positives (FP): Incorrect positive predictions of negative class instances
- False Negatives (FN): Incorrect negative predictions of positive class instances

Some classification models produce a probability score for each instance. A threshold is applied to convert these probabilities into definitive class predictions (e.g., predicting a compound as toxic or nontoxic). By adjustment of a threshold, different trade-offs can be made between capturing more true positives or avoiding false positives.

From the confusion matrix, a range of metrics can be derived to evaluate different aspects of the model’s performance, some of which we will describe in the following. Accuracy is a fundamental metric that measures the proportion of correct predictions (both true positives and true negatives) among the total predictions.
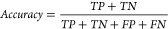


However, in contexts like drug discovery and toxicity prediction, data sets are often highly imbalanced, with significantly more inactive compounds than active ones. This imbalance poses challenges because a model could achieve high accuracy by predicting the majority class all of the time, offering little practical value. For example, if the inactive class comprises 99% of the total data set, a model solely predicting this class would be 99% correct overall, but the model would still be of no practical use (since it never predicts a compound as active for a given end point).

To address the limitations of accuracy in imbalanced data sets, metrics such as balanced accuracy becomes relevant, which consider the accuracy on the *class* level equally, i.e. this metric pays the same attention to both classes, independent of the number of data points they contain, thereby removing the above bias (while introducing of course another).
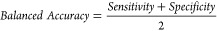


Precision measures the proportion of true positives among all positive predictions, emphasizing the model’s ability to avoid false positives, and describing the ‘trust’ (likelihood of a positive data label) for any positive prediction the model makes.
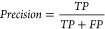


Recall (or sensitivity) assesses the proportion of true positives identified out of all actual positives, focusing on the model’s capacity to capture, to retrieve, all relevant instances.
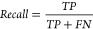


The F1 Score is the harmonic mean of precision and recall. Given its ability to account for both false positives and false negatives, the F1 score is widely used and particularly useful in imbalanced data sets. On the other hand, it does not consider the true negatives in this measure, and the same F1 score can be obtained by very different precision and recall values, and hence model behaviors. It can be seen that every metric has particular trade-offs of which aspects of model performance it pays attention to.
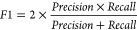


Because annotated toxicity data sets are typically smaller than in other domains, chance plays a significant role in producing hits. Cohen’s Kappa^304^ measures the agreement between actual and predicted values, adjusted for chance agreement, making it valuable for imbalanced data sets. However, it weighs predictions of the positive and negative classes equally, whereas in toxicity prediction, false positives (overpredictions) are often tolerated more than false negatives (not detecting potential toxic compounds).

**Figure 9:**
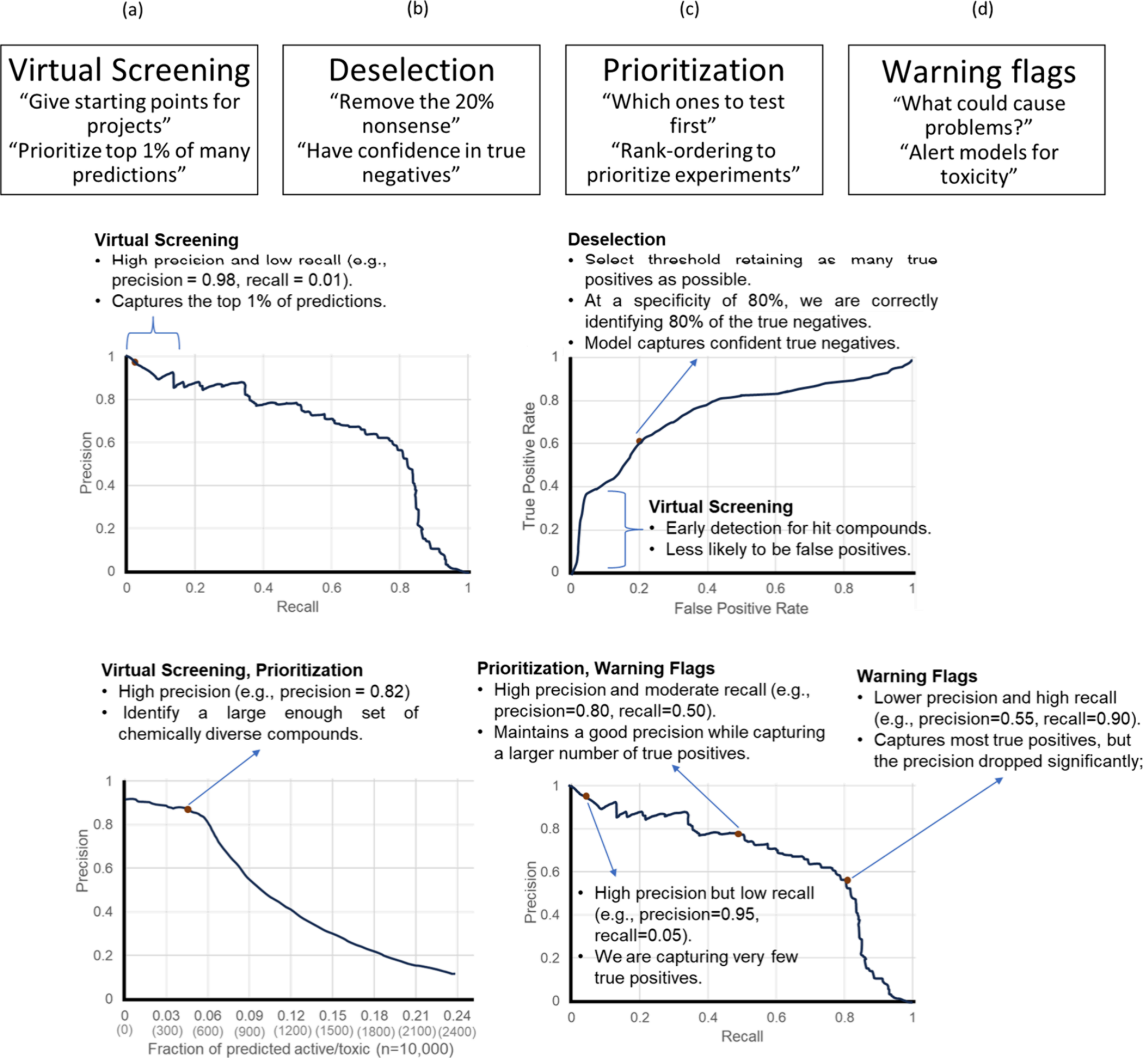
Evaluating ML model predictions in various settings: the primary goal is to prioritize specific predictions for that setting rather than rely on predicted probabilities across a range of possible thresholds. Strategies include (a) selection, (b) deselection, (c) rank-ordering, and (d) flagging toxic compounds. The choice of evaluation metrics in regression

It is also common practice to visualize model performance using Receiver Operating Characteristic (ROC) and Precision Recall (PR) curves (Figure 9). The ROC curve displays the relationship between the True Positive Rate (TPR, also known as recall or sensitivity) and the false positive rate (FPR, 1 – specificity) across various thresholds. The Area Under the ROC curve (AUC-ROC) provides a summary of the model’s overall performance. However, in imbalanced data sets, AUC-ROC can be misleading, as the model might achieve a high score simply by prioritizing the majority class. PR curves plot precision against recall at different thresholds. The Area Under the PR curve (AUC-PR) is especially useful for imbalanced data sets, as it focuses on positive class performance.

To translate predictions from models to decision making in drug discovery, it is often relevant to visually inspect the PRC and ROC curves obtained (Figure 9). Although often reported in benchmarking studies where large numbers of predictions and tasks are summarized, AUC-ROC and AUC-PR consider the *overall quality* of models based on a full range of thresholds for classification, so they are less helpful for a particular real-world use case where a particular decision needs to be made. In the real world, we may need, e.g., ML models in virtual screening (Figure 9a; early selection), removing the bottom 20% of least suitable predictions (Figure 9b; say compounds certain to be unsuitable due to high clearance), prioritizing specific predictions, such as identifying the top 1% of predictions (Figure 9c), or rank-ordering which compounds to test first (Figure 9c). For these decisions, it is often important that models should be evaluated based on the actual predictions of classes rather than model predicted probabilities.

For virtual screening^305^ (Figure 9a), what matters most for ML models is prioritizing compounds from hit triage toward validation. From an evaluation perspective, in this setting a model needs to be better at early detection (high sensitivity at low false positive rates), and the modeler needs to determine the threshold for prediction according to the practical use case, such as the number of compounds that can be selected for experimental testing.^306^ There are metrics, such as the Enrichment Factor, designed to emphasize the early recognition compared to the larger set of compounds considered in the ROC curve.^307^ Enrichment factor refers to the ratio between the proportion of true positives among the top-ranked candidates and the proportion in the full screening set for some number or fraction of top-ranked candidates. A higher enrichment factor indicates that the model is effective at early detection, identifying active compounds among the top-ranked candidates. From an AUC-ROC perspective, the enrichment factor is primarily dependent on the initial segment of the curve. It is often more practical to work with absolute numbers, such as selecting 100,000 compounds for testing (or screening 100 × 96-well plates), to align with the specific capacity or resources available. Another way to achieve these objectives is by analyzing the regions of the PRC and ROC curves. For example, ranking the top 1% of compounds predicted correctly as active (true positives), one could identify the region of the PRC curve where the highest precision is achieved, while recall is relatively able to capture 1% of positives. Typically, the top 1% of compounds would be at the beginning of the PRC (Figure 9a), typically at the top left of the curve, where the model makes predictions with the highest precision because the model is most confident about these predictions. Since it is often hard to determine what parts of the PR or ROC curves map to percentage of compounds predicted active, another way to evaluate models for prioritizing compounds would be to plot precision as a function of the percentage of compounds predicted active (with exact numbers tested on a secondary axis).^308^ Overall, we would want to identify a set of chemically diverse compounds, while a model that has high precision. To do so, modelers could choose a threshold that has acceptable precision and that is large enough to perform diversity selection.

Deselection involves setting a threshold on the ROC curve to correctly identify a large proportion of undesired compounds as true negatives while retaining as many desired compounds as true positives as possible (Figure 9b). To ensure a high recall of the undesired class (e.g., 90%), we aim to maximize true negative rate specifically subject to this constraint.

Prioritizing (or ranking) compounds based on their likelihood of being true positives involves analyzing the Precision-Recall Curve (PRC) to determine where precision and recall maximize the true positive rate (Figure 9c). The goal is to identify a set of compounds that is sufficiently large to support a selection of the desired size while also ensuring a high confirmation rate. This selection typically occurs in a region of the PRC where the model maintains good precision while recalling a significant number of true positives. An alternative way to evaluate the model’s ability to rank compounds is to plot precision as a function of the number of compounds predicted to be positive (Figure 9), which is often easier to interpret.^308^ For finer granularity, a rank-based metric such as Kendall’s tau can also be useful.

For toxicity prediction tasks, when the goal is often to flag compounds with likely toxicity, prioritizing a higher recall of toxic compounds is often more appropriate, even if it results in lower precision, which is associated with more false positives (Figure 9d), because advancing toxic compounds is highly costly. This approach is related to a deselection setting, where we aim for a high recall of desired compunds. On the other hand, if the model is used to deselect compounds, a high true negative rate (removing *mostly* toxic compounds) is desirable to preserve as many safe compounds as possible. It can be seen that models present a trade-off, and not all that is desirable can be obtained at the same time; being able to, say, have a higher recall of desired compounds comes at the cost of more false-positive predictions, etc. This underlines the importance of choosing a suitable performance metric and classification threshold for a particular use case, as opposed to trying to find a generic answer to the problem.

Models should also be compared with baseline models (such as majority class or average predictor or randomly shuffled labels using Y-Scrambling^309^), in order to establish how much value the proposed model provides. Baseline models can provide surprisingly good performance in many cases, such as on imbalanced data sets (where one can predict the majority class), or otherwise biased data sets, where the baseline classifier just picks up the underlying bias of the data set.^310^

For classification models, it is necessary to compare models to chance prediction and baseline models, in particular, given that even baseline models can obtain surprisingly good performance. Cohen’s Kappa measures the agreement between actual and predicted values, adjusted for agreement occurring by chance.^304,311^ In terms of applications, Cohen’s Kappa was used as the metric to identify the optimal decision threshold that maximizes the balanced accuracy of the classifier predicting structure–activity data from 138 public data sets corresponding to pharmaceutical targets, addressing the issue of class imbalance, where the model may overpredict the majority class and underpredict the minority class.^312^ The authors found that optimizing thresholds significantly enhances prediction outcomes for inhibitors of Tau fibril formation, particularly when the initial model is already well-predictive (as measured by AUC-ROC). This optimization improved the True Positive Rate (TPR, also known as recall and sensitivity) from 4% to 43% and Cohen’s Kappa from 0.06 to 0.4. However, for predicting inhibitors of Marburg virus binding or entry into cells, where the initial model had lower predictive power, the improvements were more modest, with TPR increasing from 0.1% to 5% and Cohen’s Kappa from 0.002 to 0.08. Where there are several classes to predict (such as low, medium, and high solubility), Kappa can be used to rank classification models; this was used in recent Kaggle solubility challenge.^313^ Overall, Kappa-based optimization is recommended for machine learning classification models in toxicity prediction, particularly when the initial model performance is strong.

Overall, the threshold chosen to generate definitive model predictions from model-predicted probabilities is context-dependent for each task. The final model should be chosen based on the desired balance between the performance metric most relevant for a particular use case.

To evaluate a regression model, it is essential to check at least three aspects of the prediction: (a) correlation—how well the model captures the relationship between the real target values and predicted data, (b) goodness of fit—how well the model fits the data overall and how much of the variance in the output variable is explained by the model, and (c) dispersion of errors—how close the predicted values are to the actual target values. Distribution- and point-based metrics are generally used to evaluate regression models.

The simplest way to evaluate a regression model is by measuring correlation, which tells us about the strength and direction of the relationship between the model’s predictions and the actual values. A common metric for this purpose is the Pearson correlation coefficient (*r*), which ranges from −1 to 1. If *r* is close to 1, it indicates a strong positive relationship, meaning that the model’s predictions closely match the actual target values. A value close to 0 (or negative) suggests little to no linear relationship or even an inverse relationship, indicating that the model may not effectively capture the trend in the data. The squared Pearson correlation (*r*^2^) measures the proportion of the variance in the actual values that is predictable from the model’s predictions. Another useful correlation metric is Spearman correlation, which is used when we are more interested in the ranking of values rather than their exact predicted values. Unlike Pearson correlation, Spearman correlation focuses on the rank order of the predictions compared to the actual values and is thus less sensitive to outlier values. This makes Spearman correlation useful for nonlinear relationships between predictions and actual target values, and in practice it can be a very useful metric to establish the ability to prioritize, e.g., compounds to be tested experimentally in an assay, where the number of compounds to select is fixed (and only depends on the ordered list of model output).

The second aspect of model evaluation is assessing the goodness of fit, which tells us how well the regression model fits the data points. There are multiple metrics commonly used to evaluate goodness of fit such as coefficient of determination (*R*^2^), coefficient of determination on regression through the origin (*R*_*o*_^2^), and modified *R*^2^ (*R*_*m*_^2^).^314,315^ An *R*^2^ value closer to 1 indicates that the model explains a large percentage of the variability in the data, suggesting a good fit. It is well established that regression through the origin (*R*_*o*_^2^) is of less significance to predictive ML models, where we wish to compare observed versus predicted values. Global metrics like *R*^2^ are often less informative because they are not measures of the decisional impact; often, errors near the decisional boundary are most important instead of equally weighting everywhere. Alexander et al. demonstrate that *R*^2^ (as defined in equation below), defined as the proportion of total variability explained by the model (the ratio of explained variability to total variability, subtracted from unity), is practically useful, particularly when the goal is to minimize residuals between model predictions and actual values.^316^
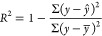
where *y* is the observed response variable, *y* is its mean, and *ŷ* is the corresponding predicted value.

While *r*^2^ and *R*^2^ are sometimes equivalent, they are not always the same. The key difference arises when the mean of the model’s predictions does not match the mean of the real data. In such cases, *R*^2^ will decrease because the model’s predictions are biased, either systematically overestimating or underestimating the targets. In contrast, *r*^2^ remains unaffected by this bias as it only captures the strength of the linear relationship between the predictions and targets. Therefore, *r*^2^ may still be high even if the model’s predictions are consistently offset. Whether to prioritize *R*^2^ or *r*^2^ depends on the goal of the model. If the primary interest is in capturing the underlying trends without regard to systematic bias, *r*^2^ may be more informative. However, if the exact accuracy of predictions is critical, *R*^2^ is more appropriate as it penalizes bias.

While metrics like the coefficient of determination (*R*^2^) are standard for evaluating how well a regression model captures the overall trend and variance of the data distribution (measure of distribution), Alexander et al. suggest that it is also important to assess the accuracy of predictions at individual data points (measure of dispersion).^316^*R*^2^ measures how well the predicted values replicate the variability of the true data but does not detect point-wise deviations where predictions are systematically higher or lower than actual values.

To capture these point-wise deviations, point-based error metrics are used that are, when it comes to anticipating the error of a prediction in future use cases, at least as important as the above distribution-based metrics. Studies often use Mean Absolute Error (MAE), which measures the average magnitude of the errors without considering their direction, offering a straightforward measure of how much, on average, the predictions deviate from the true values at each point.
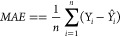
where for *n* predictions from a sample of *n* data points, *Y*_*i*_ is the observed value of the variable being predicted, and *Ŷ*_*i*_ is the predicted value.

The most common metrics used is the Mean Squared Error (MSE), which calculates the average of the squared differences between predicted and actual values at each data point. A lower MSE indicates that the model’s predictions are generally close to the actual values on a point-by-point basis.
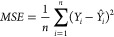
where for *n* predictions from a sample of *n* data points, *Y*_*i*_ is the observed value of the variable being predicted, and *Ŷ*_*i*_ is the predicted value.

The Root-Mean-Square Error (RMSE) is the square root of the MSE and provides an error measure in the same units as the target variable, enhancing interpretability.
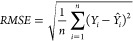
where for *n* predictions from a sample of *n* data points, *Y*_*i*_ is the observed value of the variable being predicted, and *Ŷ*_*i*_ is the predicted value.

In some regression tasks, it can be useful to evaluate model performance using classification metrics by converting continuous targets into binary labels based on a threshold. For instance, if the target values are bimodal or contain a small fraction of extreme values, we can convert them into binary or multiclass labels and compare the actual data to the predicted labels. Also in case of data with large experimental error, such as microsomal stability assays, categorical predictions based on a regression model can be useful.^317^ Metrics such as accuracy, precision, recall, and F1 score can then be used to assess the model’s ability to correctly classify target values. This approach is particularly helpful when the continuous target values are noisy within each mode, and the primary focus is on accurately making a binary decision: identifying high-risk or high-value cases.

From the perspective of ML models in toxicity prediction, a model with a low *R*^2^ may still be helpful in practical drug discovery if the measure of dispersion, such as the Root-Mean-Square Error (RMSE), is low and acceptable for the intended use. For example, *in vivo* pharmacokinetic parameters are organism-level data that are considered difficult to model using chemical descriptors: models usually have a low *R*^2^.^124,125^ Yet, such models where the RMSE is deemed within acceptable thresholds are used internally in pharmaceutical companies. In predictive *in vivo* PK property models, the number of compounds predicted within 2- or 3-fold error (prediction fold error) is a common evaluation metric.^318^ This level of ‘error’ is acceptable due to the significant inherent variability in biological systems, as well as differences in the output variable that span multiple orders of magnitude, meaning that even the experimental data can exhibit substantial variability between individuals, and predictions falling within a 2- to 3-fold range of observed values are considered reasonable in PK prediction, as the model’s performance aligns with the actual variability in the data.^319,320^

Overall, we recommend measures of dispersion like RMSE and Mean/Median Absolute Error (MAE) along with fit-to-data-distribution metrics like (*R*^2^) as metrics in evaluating toxicity models for real-world applications. (For a more detailed of those aspects, see ref (33).) In addition, it is important to keep in mind that the impact of experimental assay error should not be overlooked in the pursuit of achieving the ‘best’ performance and that model error needs to be seen in context of experimental error. Once a model reaches the known theoretical accuracy (and error) of the experimental method (the data from which the model was built on), the model could be considered as good as the underlying experiment.^321^ Striving for performance beyond that point, on the same test set, may only reflect the error and noise inherent in the data and hence lead to model overfitting (which indeed seems to be the case for several published models^322^).

## Retrospective Model Validation

**Figure 10:**
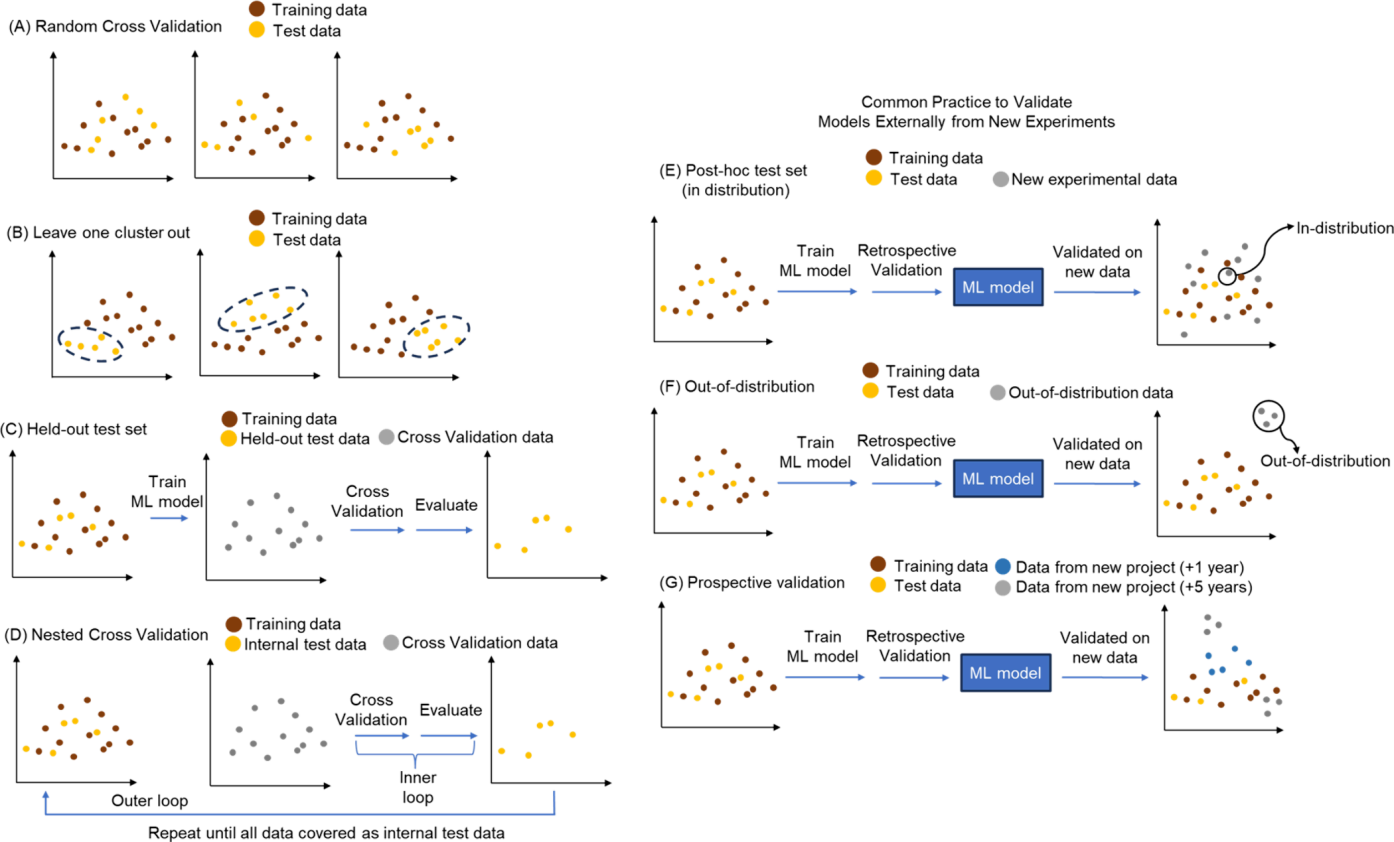
Comparative overview of model validation techniques for ML models in chemical space for toxicity prediction: Internal validation methods include (a) cross-validation, ensuring model reliability through repetitive training and testing on different data segments, (b) leave-one-cluster-out, assessing generalization across similar compound groups, (c) held-out test set, evaluating unseen data held-out from the data set; and (d) nested cross-validation, optimizing model parameters while preventing overfitting. External validation techniques comprise (e) a posthoc test set, providing an unbiased assessment of completely new experimental data; (f) an out-of-distribution set, testing model robustness against novel compound distributions such as new class compounds; and (g) prospective validation, confirming real-world applicability on future projects. Note: This figure offers an oversimplified representation of chemical space. While the 2D space can be visualized on two axes, chemical space is multidimensional, underpopulated, and behaves differently in every area. Thus, validation in chemical space is more complex than visualized here, and this figure is intended for illustrative purposes only.

In order to optimize hyperparameters of models, as well as to obtain an estimate of future performance, models must be validated on compounds not seen during model training before real-world use (see Box 2 for details on validation strategies). Models are typically validated by splitting the data to create train, validation and test sets (Figure 10), where the primary purpose of the training set is to fit model parameters; the validation set is used to optmise hyperparameters (however, preoptimized hyperparameters can also suffer from overfitting^323^) and the test set is used to estimate future model performance.

**Chart Box 2:**
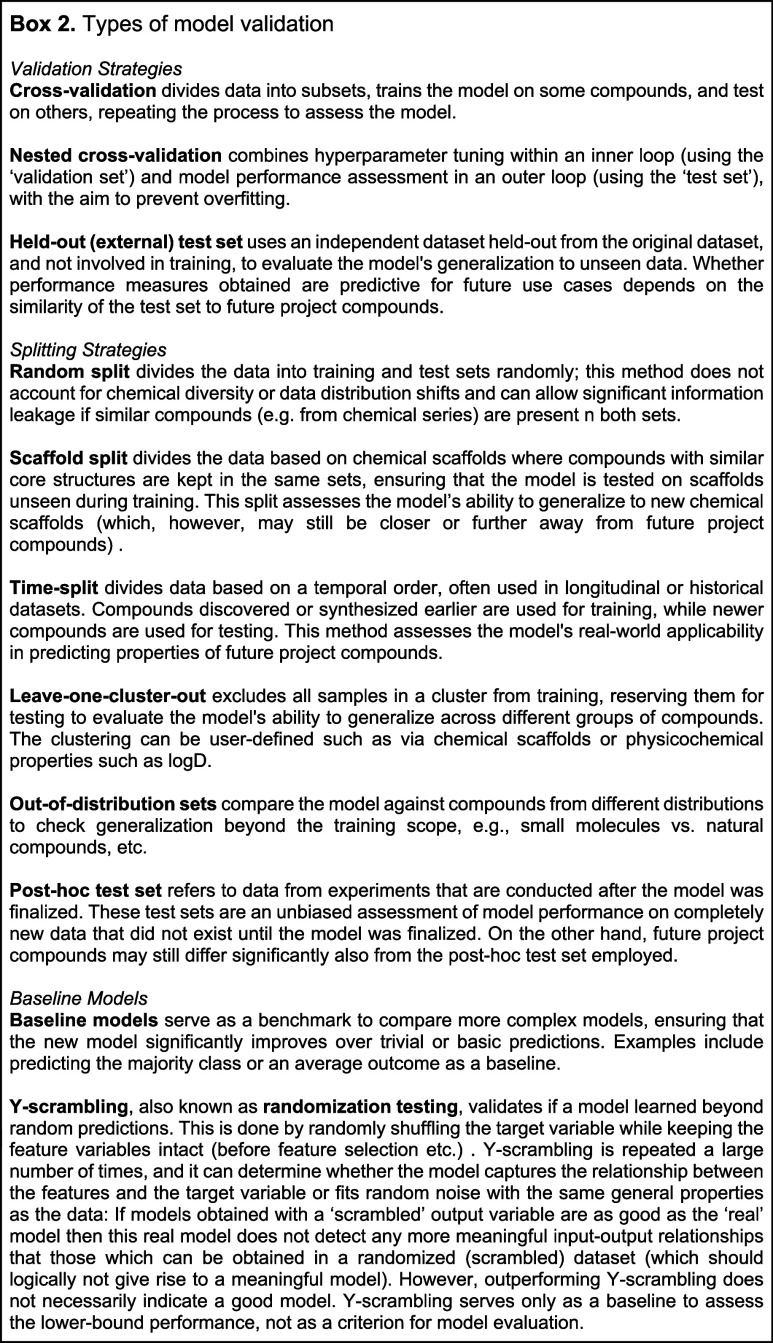


More specifically, cross-validation is often used to provide a better performance estimate than that given by a single train-test split and to estimate the uncertainty in this performance estimate. Cross-validation (Figure 10a) involves dividing the data set into multiple subsets and systematically training and validating the model across different combinations of these subsets.^234^ In each iteration, one subset of the data is used for training, and the remaining subset of the data is used for testing. Performance metrics are each averaged across splits to provide an estimate of the model’s performance, and the variance of each metric across splits is used to provide error bars for this performance estimate. If there is no need to select and optimize hyperparameters for an algorithm, then a cross-validation is sufficient. However, when optimizing parameters, a simple cross-validation often leads to an overestimation of model performance.^324^ In any case, the caveat remains that performance of the model is still evaluated only on the chemistry contained in the test set, which may not represent future use cases. Hence, nested cross-validation (Figure 10d) extends this concept by incorporating an inner loop for hyperparameter tuning (‘validation set’) and an outer loop for model performance assessment (‘test set’). This approach aims to prevent overfitting by ensuring that hyperparameter tuning does not bias the model’s performance evaluation. Once a model has been optimized with respect to hyperparameters, it is typically retrained on all training data and validated on held-out data retrospectively (Figure 10c). A held-out (‘external’) test set is where a part of the original data set is reserved only for testing a single time, ensuring that the model was never exposed to it during prior steps (such as feature selection, model selection/training, nested-cross validation, etc.). Holding out test compounds from the same source data, however, means the test data is being drawn from the same underlying distribution as the training data, which can represent ‘data leakage’ (given that similar compounds are present in training and test set) and lead to an overestimation of model performance. If a model performs similar across different folds of the (nested) cross-validation it can be seen as ‘stable’, and if it performs well on a held-out test set, the model can be considered reliable *in the respective chemical space and conditions covered by this test set* (which may or may not be predictive for future use cases, particularly if the training set and test set contained highly similar compounds, allowing information leakage, but future use cases do not). Given future use cases are generally unknown, it is difficult to obtain a reliable estimate of model performance for this situation.

There are different approaches for data splitting, which impact how the training and test sets are formed.^325^ The simplest is randomized data splitting, which assigns each data point randomly to training or test sets. Although this often still being used, it poses dangers: training data sets may differ in distribution from the data seen during model deployment—for example, the training set may contain clusters of compounds from the same chemical series, whereas the data the model is deployed on may include novel series—and thus other strategies would more realistically reflect expected model performance in real-world use.^326−328^ For instance, “scaffold splitting” groups compounds based on their molecular scaffolds—the core structures of molecules without side chains.^326^ By ensuring that compounds with the same scaffold are all in either the training or test set, metrics evaluated with this train-test split measure the model’s ability to generalize to new chemical scaffolds not seen during training. While this is conceptually valid, ‘new chemical scaffolds’ may still be more similar, or less similar, in the test set compared to the training set relative to future use cases. Related approaches split data based on other methods of clustering molecules based on various measures of similarity to avoid information leakage from training to test. A more stringent variant of data split is leave-one-cluster-out (Figure 10b) validation, where each *cluster* forms a separate test split. In effect, this splitting strategy carves out whole *areas* of chemical space for the test set. A temporal (time) split, on the other hand, involves dividing the data based on the time of data acquisition or compound synthesis. The model is trained on older data and tested on newer data, simulating real-world scenarios where models are applied to future compounds, designed based on measured properties of past compounds.^328^ This method evaluates the model’s predictive performance over time and can reveal if model performance suffers due to the underlying data distribution changing over time. This approach is meant to measure model performance on ‘new project compounds’; however, given that ‘new project compounds’ may still be located in very different areas of chemical space, this may or may not resemble future applications of the model. Step-forward splits are another approach of model validation, where data are split based on an ordering based on a molecular property, which could be a physicochemical property like logD or molecular weight. Molecules falling within some range of the property (often the highest or lowest values) are held out from training and are used for testing.^327,329^ This strategy mimics the application of a model to data that may have different properties to the training set (for example, molecules in different ranges of physicochemical space) and can thus help in understanding how the model will perform in practical, forward-looking applications. It can be summarized that various data splitting strategies for model training exist; but due to the size of chemical space and unknown future use cases, obtaining a reliable model performance estimate is difficult in practice.

## Prospective Model Validation on Relevant Chemical Space

While the above retrospective data splitting strategies can give some estimate of performance in future use cases, the real proof of applicability of a model rests in *prospective* applications; after all, it is always easier to predict the past, than to predict the future. Prospective validation involves testing the model’s predictions against new, unseen data on future projects or compounds, to confirm their reliability in the chemical space required for the project. While some of such cases are represented to model applications in true future projects, also some ‘mixed’ approaches are possible. A posthoc test set (Figure 10e) consists of validation of predictions for molecules (say, newly synthesized compounds) that the model has never encountered during any stage of its development. These molecules should be kept hidden from the model developers until the final evaluation phase. An out-of-distribution validation (Figure 10f) includes compounds that are significantly different from the training data distribution—such as new classes of compounds, novel scaffolds, or data obtained under different experimental conditions. The difference to retrospective validation is here fluent in a way, and artificially ‘difficult’ retrospective cases can be more difficult for the model to handle than, e.g., close analogues in a true prospective set. Finally, prospective validation on future projects (Figure 10g) would involve applying the model to predict outcomes in future projects or compounds synthesized after the model’s development, to test the model’s predictive power in live scenarios, reflecting its utility in actual practice. And this, in combination with what is described in the next pillar, is actually what matters in reality: Not whether a validation is ‘retrospective’ or ‘prospective’, but rather whether we can improve decision making (which of course will then usually be in the prospective domain).

The overall process of model training and validation is explained by using a hypothetical case here: Take for example, a model aiming to predict mitochondrial membrane depolarization, which will be applied in a discovery project for a novel class of chemical compounds, synthetic cannabinoids. An ML model could have been trained and internally validated using nested cross-validation on a data set for small molecules and associated mitochondrial membrane depolarization readouts (a toxicity measure); this set of compounds could include some known cannabinoids. The nesting can leave one-cluster-out to ensure that the optimization results in a model that can generalize across different subclasses of compounds, including cannabinoids. Once optimized, the fitted model should be validated retrospectively using a held-out test set, including some novel synthetic cannabinoids that were not part of the training data (out-of-distribution) to assess its predictive accuracy in the particular chemical space. If the performance is acceptable for practical application, the model could be applied to a drug discovery project targeting the development of cannabinoid-based therapeutics. A prospective validation would be attempted against experimental data from the new cannabinoid compounds developed during the project. Such prospective validation assesses whether the models perform as expected in practical project applications.

Once validated, a model can be retrained on all available data, including the test data, before it is deployed again. This should expand the model’s coverage of the chemical space since more data are included for model training. Given that the test set typically represents a small addition to a much larger training data set and the optimized model parameters remain the same as the before, the inclusion of the test data in the retraining now is unlikely to significantly alter predictions in other areas of the chemical space. Instead, it primarily enhances the model’s accuracy within the newly added chemical space. On the other hand, using the test data in training the final deployed model means that further performance evaluation of the model is difficult unless new test data becomes available. Thus, as new data become available from experiments, the model is retrained, re-evaluated, updated if suitable, and deployed for use. This process is particularly important in the case of project work, where information about the chemical space of current interest can greatly increase model performance. For an example of implementation of suitable validation protocols, see OCHEM (https://ochem.eu), a platform developed more than 15 years ago.

## Defining the Applicability Domain

Optimizing a model for a given evaluation metric needs to be complemented with a measure how well the model is able to predict for future uses cases - similar to experiments, which result, e.g., in a mean and an associated standard deviation, predictions need to come with a prediction value, and an associated confidence measure into this prediction.^330^ Models must, therefore, balance optimizing the performance metric with the so-called ‘Applicability Domain’ (AD). The AD defines the range of input/output values or conditions under which the model’s predictions are considered reliable and valid. It essentially sets the boundaries within which the model can be expected to perform accurately (to various quantitative degrees), based on the data it was trained on and the model training and validation process used. Models trained on a smaller data set of more project-relevant compounds may outperform those trained on larger data sets; this was observed in the context of virtual screening, where removing half of the molecules with the lowest applicability scores indeed *improved* performance.^331^ One approach to evaluating performance with respect to the applicability domain involves using test sets split into distance bins (where distance refers to structural similarity to the training compounds) to monitor performance. Furthermore, bins representing chemical space with poor performance suggest new experiments, whose data might improve the model performance in that space. Various alternatives have been explored to define AD in chemical space (Table 8), each catering to particular scenarios.^40^ It should be noted that while a large number of such approaches exist, given the size of chemical space and that it behaves very differently locally, it is very difficult to come with reliable error (and applicability domain) estimates in practice.

**Table 8:** Methods to Estimate the Applicability Domain of a Predictive Model for Small Molecule Compound Properties^331^

Method	Description	When to Use	When Not to Use	Reference
Range-based methods	Define boundaries based on the range of descriptor values in the training set	When the descriptor values are well-defined and bounded within a specific range	When the data contains many outliers	(40)
Convex hull methods	Establish a geometric boundary around training data points	When the data points form a well-defined area in the descriptor space	When the data is multidimensional, sparse, or irregularly distributed	(332)
Distance-based methods	Measure the distance between a query molecule and the closest training data points	When the distances between data points in the descriptor space are meaningful and consistent	When the descriptor space is a binary vector or too high-dimensional	(333)
Density-related methods	Evaluate the density of data points around a query molecule	When data consists of a few chemical series, the density of data points around a query molecule indicates prediction reliability	When the data is uniformly distributed or lacks significant density variations	(40)
One-class SVM	Train a model to identify the region in the descriptor space occupied by the training set	Identifying whether a query point belongs to the same distribution as the training set	When the training data is highly heterogeneous or contains multiple clusters	(334)
Uncertainty quantification and confidence estimation	Assess prediction confidence by analyzing model outputs	Report the confidence of the model’s predictions	When the computational cost of uncertainty estimation is prohibitively high	(335,336)
Reliability-density neighborhood approaches	Combine density and distance metrics to define AD	Combining density and distance metrics can provide a more nuanced definition of AD	When the data lacks sufficient density or distance information to make reliable assessments	(337)

## Pillar 5: Translating to Decision-Making

**Figure 11:**
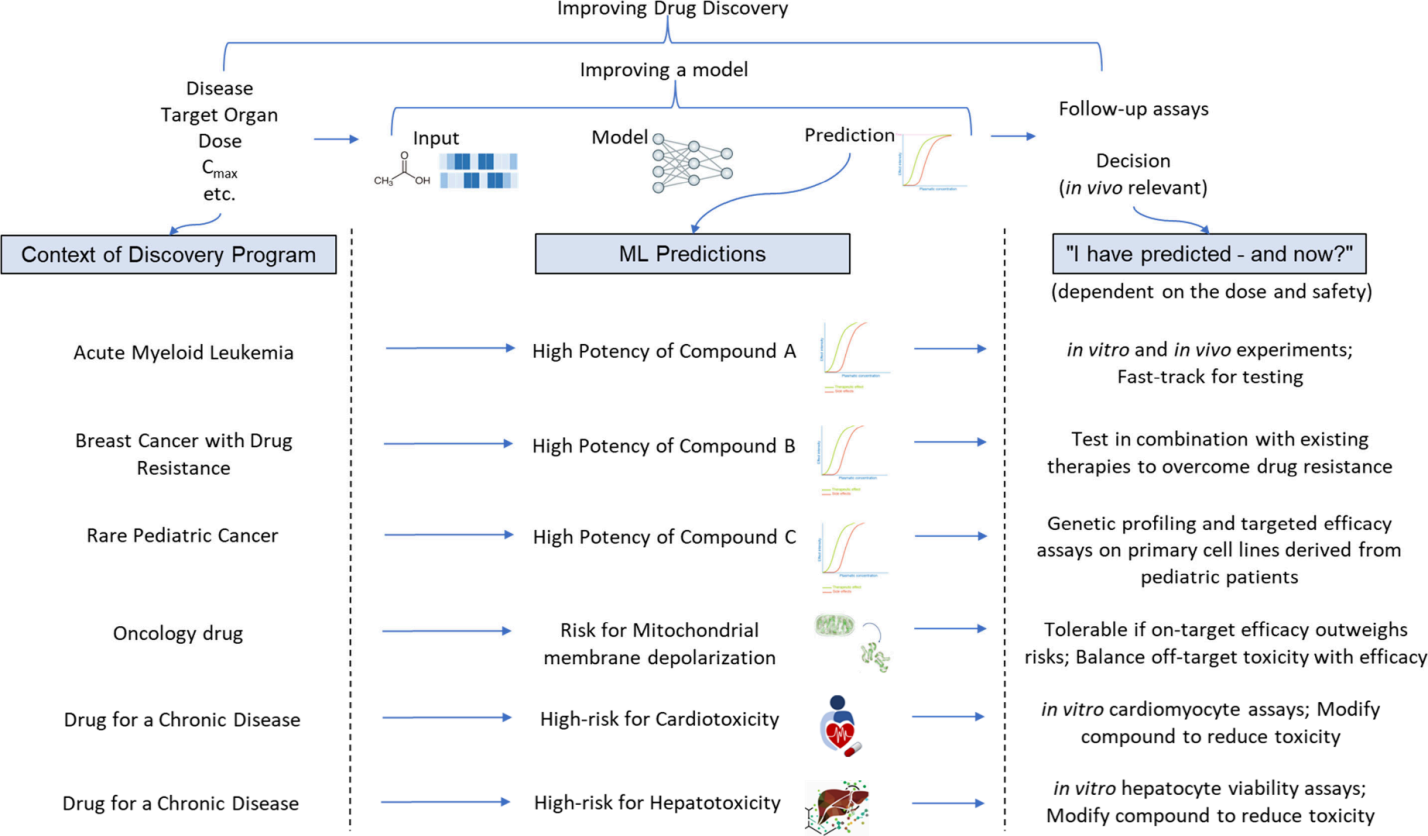
Influence on ML predictions in hypothetical project contexts depends on the availability of assays and validation on a subset of compounds. Interpreting and understanding predictions from ML models.

As we have seen, improving ML models for molecular toxicity prediction relies on data, chemical representation, model architectures, and validation. However, improving drug discovery (as a process) involves translating the outcomes of ML models into actionable follow-up decisions, such as prioritization of one compound over another, which assay to run next, etc.^300^ (Figure 11; see Box 3 for critical takeaways on moving ML predictions toward actionable drug discovery decisions). It can be seen that a machine learning model (and its application) is only a tool, an ability—when used, it is embedded into a *context*, and that context is frequently insufficiently considered when only focusing on ‘model metrics’. However, a machine learning model is not a purpose in itself; the purpose of a model is to be applied in a context.

**Chart Box 3:**
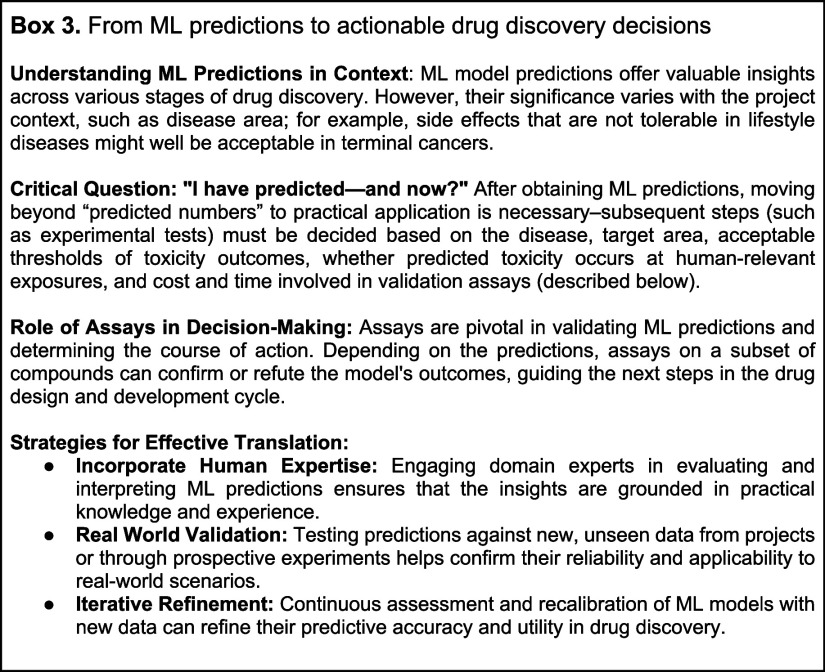


Many ML models for toxicity in the industry are used as an alert for potential risk or for prioritization and not necessarily as a go/no-go decision. Those decisions depend highly on PK/ADME, dose, exposure, etc., conditions that an ML model usually does not capture. In terms of machine learning, this represents an ‘underspecification’ of the task the model is trained on^338^—and hence the model output cannot be directly translated to decision making. For example, acetaminophen (paracetamol) is generally safe at therapeutic doses but becomes toxic at higher doses due to its metabolism into a harmful compound called N-acetyl-p-benzoquinone imine (NAPQI).^339^ Machine learning models predicting toxicity solely on the basis of molecular structure might not flag this risk because they rarely account for dose-dependent effects, metabolic pathways, or individual variations in drug metabolism (PK/ADME factors). PK parameters are needed for human-relevant decision making in safety.^340^ For example, predicting *in vivo* pharmacokinetic parameters (which cover drug absorption, distribution, and clearance in humans) could reveal unsuitable drug characteristics (such as bioavailability), informing the next iteration of drug design as part of a Design–Make–Test–Analyze cycle to optimize compounds in development.^341,342^ Overall, ML models for efficacy, toxicity, and PK/ADME can only translate to human-relevant decision-making if all three are considered in parallel.

To a small extent computational methods have already impacted regulatory processes, such as the adoption of the ICH M7 guidelines (International Council for Harmonisation of Technical Requirements for Pharmaceuticals for Human Use).^343^ These guidelines provide a framework for using computational methods, including ML, to predict the mutagenic potential of pharmaceutical impurities. The ICH M7 guidelines emphasize the importance of (Q)SAR (Quantitative Structure–Activity Relationship) methodologies, recommending two complementary approaches for this purpose, namely, an expert rule-based system and a statistics-based system. Expert rule-based systems apply established toxicological rules to identify certain molecular fragments, known as structural alerts, associated with mutagenicity, while statistical (ML) models leverage large data sets to detect patterns and predict outcomes. Where both methodologies indicate no structural alerts for mutagenicity, the guidelines deem the negative *in silico* results as sufficient evidence to conclude that the impurity is not of mutagenic concern, eliminating the need for further experimental testing. This is one concrete example of computational toxicology replacing laboratory experiments, which, however, is limited to a relatively narrow range: to *impurities* (so compounds present at relatively low concentration), in the genotoxicity area, and only when two methods are employed in parallel. In another example, French health authorities’ request for toxicity data on cyamemazine was satisfactorily answered with an *in silico* assessment of target organ toxicity.^344^ The *in silico* models used for hazard assessment covered toxicity end points such as mutagenicity, hepatotoxicity, nephrotoxicity, cardiotoxicity, etc., including two complementary models, namely expert alert and statistical QSAR models. It can be seen that model predictions can *contribute to* decision making, but having a model does not represent ‘having the answer’ by itself.

Machine learning models are increasingly being applied in real-world projects in drug discovery to uncover mechanisms of action, predict toxicity, and assess off-target risks early in the development process. Predictive assays for hepatotoxicity and cardiotoxicity have also been an essential part of the early toxicology strategy.^345^ Recently, a virtual enhanced cross-screen panel (vEXP) used ML models to predict 67 off-target activities, aiming to provide early warnings about potential adverse drug reactions.^346^ The authors reported that the vEXP panel effectively identified potential off-target activities, aiding in the early prioritization and risk assessment of drug candidates—for targets with well-balanced data sets and sufficient data points, the models demonstrated strong predictive performance, many exceeding the thresholds set for acceptable performance (e.g., Kappa ≥ 0.4, ROC-AUC ≥ 0.7). The authors suggested that the vEXP panel be integrated into various stages of the drug discovery process—to rank hit compounds based on their predicted off-target profiles, identify systematic risks associated with specific chemical series or functional groups, and ensure that late-stage optimization efforts do not inadvertently introduce new off-target activities. This example represents a case where computational models can complement experimental approaches to achieve *higher efficiency* as a result.

Extending beyond animal experiments, Next Generation Risk Assessment (NGRA) combines *in silico* models and *in vitro* assays to evaluate chemical safety without relying on animal data. For instance, an industry study on coumarin in cosmetic products integrated mathematical models, *in vitro* NAMs, an *in vitro* cell stress panel, high-throughput transcriptomics, and *in silico* alerts for genotoxicity.^347^ The authors demonstrated that *in silico* methods can complement experimental data to provide sufficient evidence for safety evaluations in a regulatory context. Similarly, Ouedraogo et al. used a 10-step framework of read-across and NAMs with propylparaben, demonstrating the practical application of integrating exposure assessment, *in silico* methods, and bioactivity data to inform reproductive toxicity alerts.^348^ Overall, these approaches highlight the value of combining multiple data sources and methodologies to enhance the accuracy, efficiency, and regulatory acceptance of risk assessments. Overall, *in silico* models used in drug (or here compound) discovery, development, and safety assessment can, to some extent, reduce the experimental burden in real-world settings.

A final key to improving the translation of ML models in the real world involves a suitable understanding of the use case and user, hence setting up a system that enables adoption in real-world projects. Aspects such as understanding the need for retraining (on project-specific data), the type of model developed (regression vs two/three/four-class classification), the output of prediction confidence (and potentially a nearest neighbor/concrete experimental data point for the user to gain trust into the model), suitable interfaces with databases and other software systems, design of the user interface, run time, etc. are important considerations in creating an impactful system in practice.^349^ Several publicly available ADMET models are commonly used (Table 9), some with web-based portals and a graphical user interface. Their ability to show feature importance, structural alerts, and other interpretive insights, where present, enhances their transparency and usability. The reader is encouraged to use these (and other available) tools to gain hands-on experience, and to see which approaches are useful in the context of their own work.

**Table 9:** Open-Source or Freely Accessible ML Models for Toxicity Prediction, with Server or Local Implementation

Tool Name	Web Site	Description	Reference
ADMETLab 3.0	admetlab3.scbdd.com	Predicts ADME-Tox properties using advanced machine learning models	(350)
QSARdb	qsardb.org	Models for human health effects, toxicokinetics, etc. for evaluating chemical and environmental risk	(352)
OCHEM	ochem.eu	Models with environmental and health related end-points, Tox21, etc.	(353)
CPSign	github.com/arosbio/cpsign	Conformal prediction for valid prediction intervals on a per-compound basis	(302)
Danish QSAR Database	qsar.food.dtu.dk	Database of QSAR models for predicting toxicological end points, especially for regulatory purposes	(354)
DILIPredictor	broad.io/DILIPredictor	Predicts human and animal-relevant liver injury and proxy liver injury end points	(355)
EPI Suite	epa.gov/tsca-screening-tools/epi-suitetm-estimation-program-interface	A suite of physical/chemical property and environmental fate estimation programs	(356)
EMolTox	xundrug.cn/moltox	Web Server for the prediction of toxicity for safety analysis in drug development	(357)
OECD QSAR Toolbox	qsartoolbox.org	Provides tools for grouping chemicals into categories and predicting their toxicological properties	(358)
OPERA	github.com/NIEHS/OPERA	A suite of QSAR models to predict physicochemical properties, environmental fate, ADME and toxicity end points	(238)
pkCSM	biosig.lab.uq.edu.au/pkcsm/prediction	Predicts pharmacokinetic properties of small molecules using graph-based signatures	(359)
PKSmart	broad.io/PKSmart	Predicts human and animal pharmacokinetic parameters	(125)
ProTox 3.0	tox.charite.de/protox3/	Virtual lab for predicting toxicities of small molecules using various predictive models	(360)
SwissADME	swissadme.ch	Predicts ADME properties of small molecules using free web tools provided by the Swiss Institute of Bioinformatics	(361)
Toxtree	toxtree.sourceforge.net	Open-source application for the estimation of toxic hazards based on decision tree approaches	(362)
VEGA-QSAR	vegahub.eu/portfolio-item/vega-qsar	Provides models for predicting toxicological end points using Quantitative Structure–Activity Relationship (QSAR) models	(363)
VenomPred 2.0	mmvsl.it/wp/venompred2	Evaluate the toxicological profile of small molecules and features that contribute to predictions to derive a structural toxicophore	(364)

**Table 10:** Commonly Used Feature/Model Importance Methods to Assess Model Performance

Method	Description	Advantages	Disadvantages	Source
Permutation Importance	Measures the increase in the model’s prediction error when a feature’s values are randomly shuffled	Easy to understand and implement; can be used in combination with any modeling method in principle	Computationally intensive and can be misleading if features are correlated	(375)
Shapley (SHAP) Value	Assigns each feature an important value based on Shapley values from cooperative game theory	Provides consistent and interpretable results	Computationally expensive, especially for complex models; can be misleading if features are correlated	(156)
LIME (Local Interpretable Model-agnostic Explanations)	Approximates the model locally with an interpretable model to explain predictions	Model-agnostic and provides local explanations	Can be unstable and sensitive to the choice of local region	(376)
Feature Importance from Tree-based Models	Measures the importance of a feature by the average gain of the splits in which it is used. Can be based on permutation feature importance or mean decrease in impurity	Directly available in tree-based models like Random Forest and XGBoost	Biased toward features with many categories	(377)
Coefficient Magnitude in Linear Models	Uses the magnitude of the coefficients in linear models to determine feature importance	Straightforward to compute and interpret	Only applicable to linear models and assumes linear relationships	(378)
Mutual Information	Measures the reduction in uncertainty about one variable given knowledge of another variable	Nonparametric and captures nonlinear relationships	Requires discretization for continuous variables and can be computationally expensive	(379)
Attention Mechanisms	Uses the attention weights in attention-based models to determine feature importance	Naturally integrated into models like transformers. Allows focusing on relevant parts of the input sequence when making predictions (captures dependencies)	Limited to models that use attention mechanisms	(289)
Counterfactual Explanations	Determines feature importance by analyzing how changing feature values affects the prediction outcome	Highlights specific changes needed to alter predictions	May generate unrealistic or nonfeasible changes	(380)
Partial Dependence Plots (PDP)	Illustrates the impact of a feature on the predicted outcome by averaging the effects of all other features.	Easy to interpret and visualize	Assumes independence of features and can be misleading if features are correlated	(381)
Prediction-Driven Molecular Matched Pairs	Examines pairs of similar molecules to identify transformations affecting specific properties.	Overcomes the data limitation problem and allows interpreting the model itself and not the experimental data	Limited number of molecules can be checked in this transformation-driven approach	(382)

Interpreting and hence, to an extent, ‘understanding’ ML models, in terms of their underlying basis for predictions, can make predictions more trustworthy and help detect biases in the model, as well as ensure the model recovers previous knowledge (e.g., known structural alerts). Suitable feature spaces and modeling architectures need to be used to interpret models, which can lead to novel understanding of the model (and problem) at hand. Feature importance measures (Table 10) can be used to estimate the contribution of individual features, particularly where the features are interpretable, e.g., specific molecular targets, such as proteins, or chemical substructures associated with toxicity, etc. For example, mutagenicity and skin sensitization are often concerns in drug development that can be interpreted from chemical features. Specific structural alerts—such as nitroaromatic groups for mutagenicity,^365^ or electrophilic groups like α,β-unsaturated carbonyls (Michael acceptors) for skin sensitization^366^—are associated with these toxicities; predictive models can identify these fragments and enable medicinal chemists to modify molecules by eliminating or altering these groups to reduce the risk (although modifying a few fragments with the highest contribution in the model does not necessarily resolve toxicity completely^355,367^).

However, interpretations are not always straightforward, especially when it comes to predictions based on the chemical structure. Misinterpretations are common for data sets with high bias in chemical space, which is often the case due to project, synthesis, and analogue bias, etc. For example, a previous study by one of the authors found that *sugar* rings were associated with the *bitterness* of compounds, a result that does not make sense at face value. In this case, the confounding factor was that natural products are frequently glycosylated *and* tend to be bitter. The model then proposed this *confounding factor* (and bias) in the model as a correlation when it was not, in fact, the *causation*.^368^ This example shows that while feature importance measures can provide actionable insights, their application in real-world drug discovery must account for potential biases in the input data.

Mechanistic insights can also be gained by interpreting ML models. For example, fuzzy rules are a set of “if–then” statements based on fuzzy logic.^369^ Unlike traditional binary logic that deals strictly with true or false values, fuzzy logic allows for reasoning with uncertain or imprecise information by accommodating degrees of truth. For example, a rule might state, “If the daily dose of an oral medication is high and the lipophilicity is high, then the probability of hepatotoxicity is high”,^370^ where the term “high” is not an exact value but a linguistic variable represented by membership functions in a fuzzy set. After training an ML model, fuzzy set rules can be developed based on how certain input features influence the model’s predictions. For example, converting decision tree rules or clustering results into fuzzy rules can make them more interpretable. For example, Friederichs et al. were able to cluster 90 environmental compounds using fuzzy clustering based on physicochemical parameters, reasoning why compounds in specific clusters exhibit aquatic toxicity (volatility, hydrolysis, etc.).^371^ Fuzzy rule sets are usually based on well-defined and interpretable variables (such as molecular weight, logD, etc.). They can help elucidate the fundamental properties contributing to toxicity and guide the design of safer compounds, where interpretability from a medicinal chemistry perspective drives decision-making.^372−374^

When interpreting model predictions, however, correlation must not be confused with causation; the most-contributing features used by the model are only meaningful if they represent the underlying cause (which is often unknown), and in practice, feature importance often suffers from a ‘long tail’ distribution, where a large number of features are associated with a prediction; this complicates interpretation (many features contribute ‘somewhat’ to the end point the model predicts). Moreover, feature importance is a local property for nonlinear models, while global for linear models. Combining modeling and computational analysis with expert knowledge is, hence, key. For example, pathway enrichment analysis involves mapping important features, such as genes and proteins, to known biological pathways using tools like KEGG, Reactome or Ingenuity Pathways Analysis (IPA); for example, Antazak et al. identified 21 molecular pathways differentially modulated in response to nephrotoxic vs nontoxic compounds.^383^ They identified the three main functional categories of pathways for nephrotoxicity—metabolic pathways (e.g., Glycerophospholipid metabolism), signaling pathways (e.g., Parkinson’s disease), and cell communication pathways (e.g., cell communication). Network analysis can extend mechanistic analysis by constructing and analyzing biological networks, such as Protein–Protein Interaction (PPI) networks, to pinpoint key nodes and modules. For example, IPA has very specific features to evaluate protein–protein interactions, such as upstream regulator analysis, mechanistic networks, causal network analysis, and downstream effects analysis.^384^ A study using IPA revealed a novel antitumor mechanism for MK886, a leukotriene antagonist, involving cytoskeleton-induced alteration of chromatin structure, revealing unknown aspects of the action and safety.^385^ Another study used PPI networks to determine targets for cardiac disorders and found protein ERBB4 interacting with known drug targets.^386^ Although ERBB4 was not a known drug target for cardiac failure at the time (according to Huang et al.^386^), there is considerable work now on using ERBB4 agonists for treating heart failure.^387^ Overall, pathway enrichment analysis and PPI networks can highlight important proteins or groups of proteins that may contribute to specific properties, yielding a deeper understanding of the complex biological interactions involved. In the cases discussed here, prior knowledge (in terms of biological pathways) was combined with data (from the models) to arrive at an interpretable model output.

Another related approach to interpretable predictions is to use causal and mathematical mechanistic models to directly establish cause-and-effect relationships rather than correlations captured by ML models. Causal inference methods have been applied directly to transcriptional data and PPI networks to distinguish correlation from causation and infer potential mechanisms of actions that drive toxicity. For example, these methods were shown to recapitulate specific pathways downstream of the molecular targets, providing a systems-level view of the drug’s mechanism.^388,389^

Overall, understanding the features involved in the predictions of ML models can provide both insight and confidence into the predictions being made.

## Pharmacokinetics in Decision-Making

Toxicity is not a simple ‘yes’ or ‘no’ question—even water can be toxic at ‘too high a dose’.^390^ Concentration and exposure (and PK in general) are absolutely critical for assessing safety and toxicity but are relatively neglected in the ML community.

Pharmacokinetics is a critical aspect of drug development and clinical decision-making because it provides detailed insights into how a drug is absorbed, distributed, metabolized, and eliminated within the body.^391^ This information is essential for determining the optimal dosage and administration schedule to achieve therapeutic efficacy without causing harm. By studying pharmacokinetics, researchers can establish the time it takes for a drug to reach therapeutic levels, how long it remains effective, and the dynamics of its elimination. This knowledge ensures that drug regimens are optimized for maximum benefit while minimizing the risk of adverse effects. Analogous considerations apply to compound toxicity.

Pharmacokinetics also plays a central role in balancing efficacy and safety by informing the therapeutic index (TI),^340^ a critical parameter derived from pharmacokinetics and pharmacodynamic (PD) studies and defined as the ratio between the toxic dose and the effective dose of a drug:
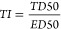
where *TD50* is the dose that produces toxicity in 50% of the population, and *ED50* is the dose that is effective in 50% of the population.^340^ A higher TI indicates a greater margin of safety between therapeutic and toxic doses. Accurate PK modeling helps to determine the TI more precisely, aiding in optimizing dosing regimens to maximize efficacy while minimizing toxicity.

While physiologically based Pharmacokinetics (PBPK) models have been used in drug discovery for a number of years, they require tailoring to the particular compound properties at hand. On the other hand, in recent years approaches have become popular to predict *in vivo* PK directly based on chemical structure,^392^ owing to the availability of suitable data sets. Machine Learning models have recently been integrated with mechanistic models to enhance the predictive accuracy. Tools that predict pharmacokinetics parameters using ML algorithms, such as PKSmart,^125^ can process large data sets to predict the pharmacokinetics parameters that can then be used as input parameters to a compartment model.^393−396^ This hybrid approach combines the strengths of data-driven ML models with the interpretability of the mechanistic models.

## Optimizing ML Strategies in Various Stages of Drug Discovery

Using ML for QSAR/QSPR models is common practice in drug discovery, where finding the right balance between cost, speed, and predictivity of a method at a given stage of the drug discovery process is critical (see Table 11). It is important to recognize that different industries may adopt varying approaches to using ML models, both within drug discovery and development and in other industries such as consumer goods or agrochemistry. In these contexts, different properties of data sets, representations, models, validation methods, and evaluation metrics may hold varying degrees of importance. Some projects might favor a balanced approach between recall and precision, while in other cases one or the other might be more important.^397^ Others may tailor their model optimization strategies (or loss functions) to particular aspects of model performance, specific therapeutic areas, the types of compounds being studied, or the technologies employed.^32,398^ A typical use of models along the early and late drug discovery stages follows.

**Table 11:** Different Priorities and Criteria Are Used in ML Models to Balance Efficacy, Toxicity, and Selection (e.g., on-target activity, efficacy) or Deselection (e.g., toxicity alert models) in Early- and Late-Stage Drug Discovery

Focus	Early Stage Exploratory/Discovery (usually qualitative)	Late Stage Refinement/Development (usually quantitative: exposure, PK, etc.)
ML models for selection (e.g., on-target activity, efficacy)	Broad selection, high recall. Include as many potential compounds fitting the target product profile as possible.	Narrow selection, high specificity. Only compounds that meet strict efficacy and safety criteria are selected for further development.
ML models for deselection (e.g., toxicity alert models)	Low impact of compounds incorrectly predicted as toxic (false positives). It is acceptable to have some false positive predictions.	High precision is desired in ML models to minimize compounds incorrectly predicted as nontoxic (false negatives), avoiding resource wastage on failing compounds.

For early stage virtual screening of compounds across a range of targets, discovery phases are exploratory, often targeting novel or less well-understood pathways. Given that on-target effects are crucial for compound selection (after a target has been selected), ideal ML models would find many hits with a high precision (i.e., with a high confidence that the compounds selected are indeed hitting the target the project is working on).^399^ On the other hand, from a toxicity prediction perspective, early discovery phases also need to *minimize compounds incorrectly predicted as nontoxic* (‘false negatives’, where toxicity is seen as the ‘positive’ label, from the modeling perspective).^345^ The above decisions depend on the project context—for example, when the project aims at conditions with limited patient survival (e.g., advance stage oncology or rare diseases), toxicity concerns may not be a major roadblock.^400^ Instead, other factors such as the compound’s pharmacokinetic profile (if distribution to target organs such as the brain is required) may take center stage. One possible strategy for using data and machine learning in a drug discovery project is visualized in Box 4. Variations need to be applied according to project needs in a given concrete situation.

**Chart Box 4:**
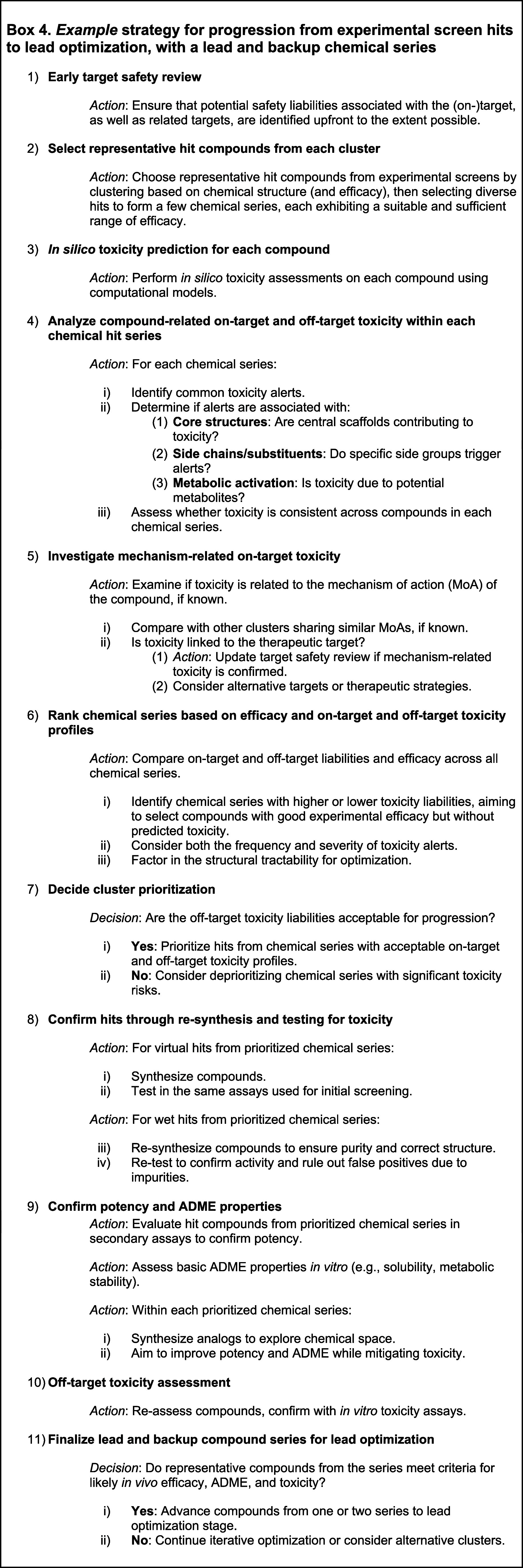


Me-too/Me-better drugs share the same mechanism of action or target as the existing drugs. Projects focused on identifying me-too drugs generally have a lower human safety risk profile because their mechanisms of action and on-target toxicity are often already known. Still, off-target toxicity may present challenges, where the adverse reactions are not caused by the therapeutic class.^401^ The opportunity also lies in finding incremental improvements in therapeutic action and/or safety. Projects focusing on me-too/follow-on drugs usually demand fewer resources to develop^402^ and thus ML models could be optimized on minimizing compounds incorrectly predicted as inactive (false negatives) among the known target (in line with the practice of revisiting prior screening data and ‘rescuing’ false negatives).^401^ To ensure competitiveness in the market, these projects would likely want to find all possible molecules in the early stage (high recall), advancing the most promising ones to development and keeping the others as backup in case the molecule fails in the preclinical or clinical stage.^403,404^

By contrast, first-in-class drugs have a new and unique mechanism of action that could yield greater efficacy than existing treatments, but carry a higher risk due to unproven mechanisms that may not work as expected, or may have unforeseen side effects or consequences.^405^ That said, a study by Health Canada considering drugs that were approved and later withdrawn found that first-in-class drugs do not exhibit a higher concern for safety compared to nonfirst-in-class drugs.^406^ Projects aiming for a first-in-class drug also offer higher rewards, with market exclusivity initially, and in later years increased market share when other pharmaceuticals enter the space (even when new molecules may have benefits).^402^ First-in-class drugs have limited or no data in the chemical space and therefore rely more on chemical space exploration. ML models employed in early discovery would ideally need to rank compounds with the desired target profile (efficacy) and low toxicity (at human exposure levels) for experimental validation.^306^

In the early screening paradigm, *in vitro* end points are relatively inexpensive to test; lead compounds that are predicted positive could be screened in those assays to detect false positives. False negatives may not get tested as they were deemed “safe” and carried forward an unidentified liability. ML models thus need to have a lower tolerance for false negatives; we would like to flag compounds if they are toxic. As a project progresses to late-stage discovery, the focus shifts to precision to ensure that only the most promising, efficacious, and safe compounds are pursued from the pool of candidates.

In summary, to translate the benefits of toxicity models to decision-making, they must consider a number of aspects in the context of the project-relevant data, suitable representations, proper validation, reliable interpretation.^127^ Given the complexity of the task, the lack of data in many domains, and the variability of the biological systems many end points are measured in, it is not trivial to get all of those aspects right.

## Conclusion

This review summarizes key challenges and considerations in translating machine learning models into decision-making tools for real-world drug discovery projects, in particular, related to compound toxicity and safety. This includes making choices about data, modeling, validation, model metrics, and applying the *model* thus obtained to the *process* of drug discovery. We hope that the reader finds this review useful and that it helps this translation from concept to practice.
